# Changes in electromyographic activity, mechanical power, and relaxation rates following inspiratory ribcage muscle fatigue

**DOI:** 10.1038/s41598-021-92060-y

**Published:** 2021-06-14

**Authors:** Antonio Sarmento, Guilherme Fregonezi, Maria Lira, Layana Marques, Francesca Pennati, Vanessa Resqueti, Andrea Aliverti

**Affiliations:** 1 PneumoCardioVascular Laboratory - Hospital Universitário Onofre Lopes, Empresa Brasileira de Serviços Hospitalares (EBSERH) and Laboratório de Inovação Tecnológica Em Reabilitação, Departamento de Fisioterapia, Universidade Federal Do Rio Grande Do Norte, Natal, Brazil; 2 Dipartimento Di Elettronica, Informazione E Bioingegneria, Politecnico Di Milano, Milan, Italy

**Keywords:** Physiology, Respiration

## Abstract

Muscle fatigue is a complex phenomenon enclosing various mechanisms. Despite technological advances, these mechanisms are still not fully understood in vivo. Here, simultaneous measurements of pressure, volume, and ribcage inspiratory muscle activity were performed non-invasively during fatigue (inspiratory threshold valve set at 70% of maximal inspiratory pressure) and recovery to verify if inspiratory ribcage muscle fatigue (1) leads to slowing of contraction and relaxation properties of ribcage muscles and (2) alters median frequency and high-to-low frequency ratio (H/L). During the fatigue protocol, sternocleidomastoid showed the fastest decrease in median frequency and slowest decrease in H/L. Fatigue was also characterized by a reduction in the relative power of the high-frequency and increase of the low-frequency. During recovery, changes in mechanical power were due to changes in shortening velocity with long-lasting reduction in pressure generation, and slowing of relaxation [i.e., tau (τ), half-relaxation time (½RT), and maximum relaxation rate (MRR)] was observed with no significant changes in contractile properties. Recovery of median frequency was faster than H/L, and relaxation rates correlated with shortening velocity and mechanical power of inspiratory ribcage muscles; however, with different time courses. Time constant of the inspiratory ribcage muscles during fatigue and recovery is not uniform (i.e., different inspiratory muscles may have different underlying mechanisms of fatigue), and MRR, ½RT, and τ are not only useful predictors of inspiratory ribcage muscle recovery but may also share common underlying mechanisms with shortening velocity.

## Introduction

Muscle fatigue is defined as the loss of capacity to develop force and/or velocity of shortening of a muscle that is reversible at rest^1^. Consequently, respiratory muscle fatigue may limit exercise tolerance, alter breathing mechanics, enhance the sensation of dyspnea, and contribute to ventilatory failure^2^.

In healthy individuals, respiratory muscle fatigue can be developed selectively either in the diaphragm or accessory ribcage muscles, depending on the recruitment pattern. Inspiratory ribcage and diaphragm fatigue can also be discriminated using twitch transdiaphragmatic pressure during cervical nerve stimulation in response to inspiratory threshold loads^3,4^. Although global inspiratory muscle fatigue (i.e., ribcage and diaphragm muscles) has been extensively studied^5,6^, selective fatigue and recovery of ribcage muscles are considerably unknown during loaded breathing^7^. During this type of contraction, continuous monitoring of local muscle fatigue is possible by measuring myoelectric activity in the frequency domain (i.e., power density spectrum) using surface electromyography (sEMG). sEMG power spectrum shifts toward lower frequencies during muscle fatigue, resulting in rapid decrease in high-frequency band, progressive increase in low-frequency band, and fall in median frequency and high-to-low frequency ratio (H/L) parameters^8,9^.

According to Allen et al*.*^10^, most studies involving fatigue are on isolated animal tissues, and an important challenge is to identify the mechanisms contributing to fatigue and recovery in humans under different circumstances. For this, experiments and apparatus must be the least painful and invasive, respectively, as possible. Conveniently, these kinds of measurements are already possible in the field of respiratory physiology. Optoelectronic plethysmography (OEP) measures chest wall and compartmental volumes using retroreflective markers placed on anatomical reference sites of ribcage and abdomen. The volume measured by OEP, without the use of any noseclips or mouthpieces^11^, can be considered an index of overall inspiratory and expiratory muscle length^12^, and shortening velocity can be accurately estimated^13^. Additionally, from the non-invasive perspective, pressure and contraction and relaxation rates obtained from nasal sniffs accurately reflect esophageal pressure curves, providing a quantitative response index to inspiratory muscle fatigue and recovery^14^. As pressure and shortening velocity are known, mechanical power of respiratory muscles can be estimated non-invasively^13,15^.

In this context, the use of only non-invasive devices and simultaneous assessment of power spectrum sEMG changes during dynamically loaded contractions may help understand respiratory muscles fatigue development and recovery in vivo. Overt muscle fatigue is preceded by several muscle changes^10^, resulting in slowing of relaxation and reduction of peak tension, maximal velocity of shortening, and power characteristics^16,17^. Although decreased shortening velocity would be the main contributor to decreases in inspiratory ribcage mechanical power, more recent literature did not show any indication of contractile slowing induced by fatigue or acidosis^18^. Thus, selective inspiratory ribcage muscle fatigue would lead to reducing force, slowing muscle contraction, modifying relaxation properties, or a combination of the three. As fatigue is related to muscle fiber constituency^19^, and this feature is measured in terms of how rapidly EMG spectral variables are reduced as a function of contraction duration^20^, median frequency and H/L of the ribcage muscles would also change with different time constants during fatigue and recovery. In the present study, we aimed to investigate contractile and relaxation properties, shortening velocity indexes, mechanical power, and sEMG changes of three inspiratory ribcage muscles before (pre-fatigue) and after (recovery) a fatigue protocol using 70% of individuals’ maximal inspiratory pressure. Experiments were performed in healthy adults, and associations between changes in relaxation rates and shortening velocity and mechanical power were investigated to observe if these parameters share a common underlying mechanism during recovery from fatigue. Relationships were also calculated with power spectrum parameters to understand whether relaxation rates were useful predictors of recovery.

## Methods

### Ethical approval

This study was conducted according to the Declaration of Helsinki and approved by the research ethics committee of the Hospital Universitário Onofre Lopes (HUOL/EBSERH—Brazil) (number 3.084.956). All individuals gave written, signed, and informed consent before participating in the study.

### Individuals

All individuals involved were laboratory personnel trained in respiratory maneuvers, self-reported healthy without nasal congestion, influenza, known septum deviation, and with no history of smoking, heart, or lung disease. Those who presented forced vital capacity and forced expiratory volume in the first second < 80% of predicted were excluded.

### Pulmonary function

Spirometry was performed using a KoKo Digidoser spirometer (nSpire Health, Longmont-USA). Forced vital capacity, forced expired volume in the first second, and peak expiratory flow were assessed according to acceptability and reproducibility criteria^21^, and highest values obtained were compared with reference values^22^.

### Respiratory muscle strength

A digital manometer (NEPEB-LabCare, Belo Horizonte-Brazil) was used to assess respiratory muscle strength by measuring maximal inspiratory and expiratory pressures^21^. Sniff nasal inspiratory pressure (SNIP) was also used to assess inspiratory muscle strength. The highest value obtained for each of the above tests^21^ was compared with reference values^23,24^ and considered for statistical analysis.

#### Sniff curve analysis

All sniffs were performed from functional residual capacity and with individuals seated on a chair without back support. The level of functional capacity was controlled by visual inspection and confirmed by OEP analysis. Contractile and relaxation parameters and contraction-relaxation coupling of inspiratory muscles were extracted from sniff traces by custom software developed in MATLAB (The MathWorksInc, Natick, MA).

The following contractile parameters were calculated: contraction time (ms), as the time to reach peak pressure^25^; maximum rate of pressure development (MRPD, cmH_2_O·ms^-1^), as the negative peak of the first derivative of the pressure–time curve; MRPD normalized to sniff peak pressure (MRPD/Peak, ms^−1^)^26^; and time to peak shortening (ms), as the time to reach MRPD^27^.

Regarding relaxation parameters, the following were calculated: half-relaxation time (½RT, ms), as the half-time of the relaxation curve; and maximal relaxation rate (MRR, ms^-1^), as the positive peak of the first derivative of pressure–time curve normalized to sniff peak pressure^28^. Time constant (τ, tau) of the later monoexponential phase of pressure decay (over 50 to 70% of pressure decay curve) was also calculated (y = exp^-t/τ^). Correlation coefficient of the individual exponential regression had to be ≥ 0.98 for a measure of τ to be accepted^29^. Contraction-relaxation coupling was assessed as the ratio between MRPD and MRR (MRPD/MRR)^27^.

The following criteria were used to select those sniffs suitable for analysis: (1) sniff performed from functional residual capacity, (2) peak pressure maintained for less than 50 ms, (3) duration of inspiratory effort less than 500 ms, and (4) sniff pressure waveform with smooth decay curve^30^.

### Optoelectronic plethysmography

Chest wall and compartmental volumes were measured by an OEP system (BTS, Milano-Italy). Eight photosensitive cameras positioned around the subject (4 in the anterior and 4 in the posterior region) captured the movement of 89 reflexive markers positioned at specific anatomical points of the thorax and abdomen. Once coordinates of the markers were acquired, a closed surface was defined by connecting the points to form triangles, and volume enclosed by the surface was calculated using the Gauss’ theorem^11^.

*OEP analysis*. Volume variation of chest wall (ΔV_CW_) and its compartments [pulmonary rib cage (ΔV_RCp_), abdominal rib cage (ΔV_RCa_), and abdomen (ΔV_AB_)] and inspiratory time (Ti) during sniffs were calculated. ΔV_AB_/Ti, ΔV_RCp_/Ti, and ΔV_CW_/Ti were calculated as shortening velocity index of diaphragm, inspiratory ribcage, and global inspiratory muscles, respectively^13^. As the product of pressure and shortening velocity can be used as an index of mechanical power of inspiratory muscles^15^, diaphragmatic (Ẇ_di_), inspiratory ribcage (Ẇ_rcm_), and global inspiratory (Ẇ_insp_) mechanical power were calculated as the ratio of SNIP peak pressure and ΔV_AB_/Ti, ΔV_RCp_/Ti, and ΔV_CW_/Ti, respectively^13^. End-expiratory chest wall volumes were also recorded immediately at initiation of SNIP maneuvers as a measure of functional residual capacity^31,32^ to verify if differences in muscle length contributed to fatigue response.

### Surface electromyography

A TeleMyo DTS Desk Receiver electromyograph (Noraxon, Scottsdale-USA) acquired myoelectric signals using 4 wireless sensors with 16-bit resolution and common-mode rejection ratio > 100 dB (Noraxon, Scottsdale-USA). Sampling frequency of the captured signals was 1500 Hz, with 500 Hz low pass filter and pre-amplification of 1000 times.

Ag/AgCl bipolar surface electrodes were placed along muscle fibers on the right side of the body to minimize cardiac electrical signals contamination. The skin was prepared to reduce impedance, interference, and noise, and all recommended procedures were strictly followed during signal acquisition^33^. Of note, electrodes were placed without rearranging placement location of OEP reflective markers. Electrodes were placed over the sternocleidomastoid (SCM), scalene (ESC), parasternal (PARA), and rectus abdominis (RA) muscles^34,35^. AB muscle activity was included to verify if true muscle relaxation accompanied the descending portion of sniff curves^36^. If active expiration was present (i.e., abdominal contraction), the sniff curve was excluded from the analysis.

#### sEMG processing and analysis

All sEMG analyses were performed off-line using the MATLAB environment (The MathWorksInc, Natick, MA). Recorded sEMG signals were preprocessed using a 20–400 Hz Butterworth bandpass filter. sEMG analyses were performed in both time and frequency domains by calculating root mean square (RMS) amplitude and power spectrum density distribution, respectively. RMS was interpreted as the magnitude of muscle activation (or excitation), and results were neither associated with muscle force^37^ nor reported as index of muscle fatigue due to its low validity during dynamic contractions^38^.

Power spectral density was calculated by applying continuous wavelet transform technique using Daubechies4 mother wavelet^39,40^. Median frequency was calculated for each power spectrum as the frequency value dividing the sEMG signal spectrum into two parts of equal energy. Additionally, relative contributions of the high- and low-frequencies to sEMG signals were estimated by filtering the signal with different bandpass filters and integrating each filter output to obtain high-frequency (H, 130–250 Hz) and low-frequency (L, 30–50 Hz) powers^41^. H/L ratio was also computed.

Lastly, as SCM, ESC, and PARA muscles contribute most to ribcage motion during loaded breathing^42^, each sEMG parameter obtained from these muscles was averaged and interpreted as electrical activity of the ribcage inspiratory muscles (rcm).

### Study protocol

All measurements were performed in a laboratory with temperature-controlled between 26 and 28 °C. All individuals performed at least fifteen SNIP maneuvers ten minutes before data collection due to learning effect of SNIP maneuver^43^. Right after, the study protocol was initiated as follows:

#### Pre-fatigue phase

The same experienced physiologist positioned the sEMG electrodes and OEP retroreflective markers while individuals remained seated on the chair. The manometer plug was inserted in one nostril (the contralateral one remained unobstructed), and individuals were asked to perform 10 SNIP maneuvers with intervals of 30 s in between. For each subject, the SNIP maneuver that generated the highest peak pressure value was defined as the pre-fatigue sniff curve^18^.

During this phase, each portion of the sEMG signal corresponding to one SNIP maneuver was submitted to time and frequency domain analyses. Mean value of each SNIP, sEMG, and OEP parameters across all individuals was used as reference value (pre-fatigue values).

#### Fatigue protocol

After obtaining pre-fatigue values, the subject remained seated at rest for 15 min while instructions about the fatigue protocol were given. The fatigue protocol was performed using a two-way inspiratory threshold valve (POWERbreathe Classic IMT, Warwickshire-UK). Although studies demonstrate that inspiratory muscle fatigue can occur at loads < 60% maximal inspiratory pressure^43^, ‘optimal’ inspiratory resistive load appears between 60 and 80% of maximal inspiratory pressure with duty cycle of 0.4–0.7^44^. Here, individuals were required to generate an inspiratory pressure sufficient to overcome a threshold load set at 70% of maximal inspiratory pressure during inspiration; expiration was unimpeded. Individuals were also instructed to recruit mainly ribcage muscles and select whatever tidal volume, respiratory rate, and breathing pattern they found necessary to overcome the load and open the valve^45^. During this phase, only sEMG signals were acquired, and the task terminated when individuals failed to generate sufficient inspiratory pressure to open the valve for three consecutive breaths or missed three consecutive breaths despite verbal encouragement. At this point, time to task failure (T_lim_) was recorded. Vocal encouragement was given throughout the fatiguing task.

For analysis, median frequency, H, L, H/L ratio, and RMS were normalized for each patient by expressing them relative to values obtained at the beginning of the fatigue protocol (i.e., mean of the initial 10 s) and plotted as a function of the total time of the run. The wavelet algorithm was applied to the overall sEMG signal, while RMS, wavelet coefficients of median frequency, H, L, and H/L ratio were analyzed in time windows of 5 s length.

#### Recovery phase

When T_lim_ was reached, individuals immediately took off the mouthpiece, placed the manometer plug in the same previously used nostril, and performed a series of 10 maximal sniffs from functional residual capacity with intervals of 30 s in between. The series of 10 SNIP maneuvers should fit criteria mentioned above^30^ to be suitable for analysis and included in the study.

**Figure 1:**
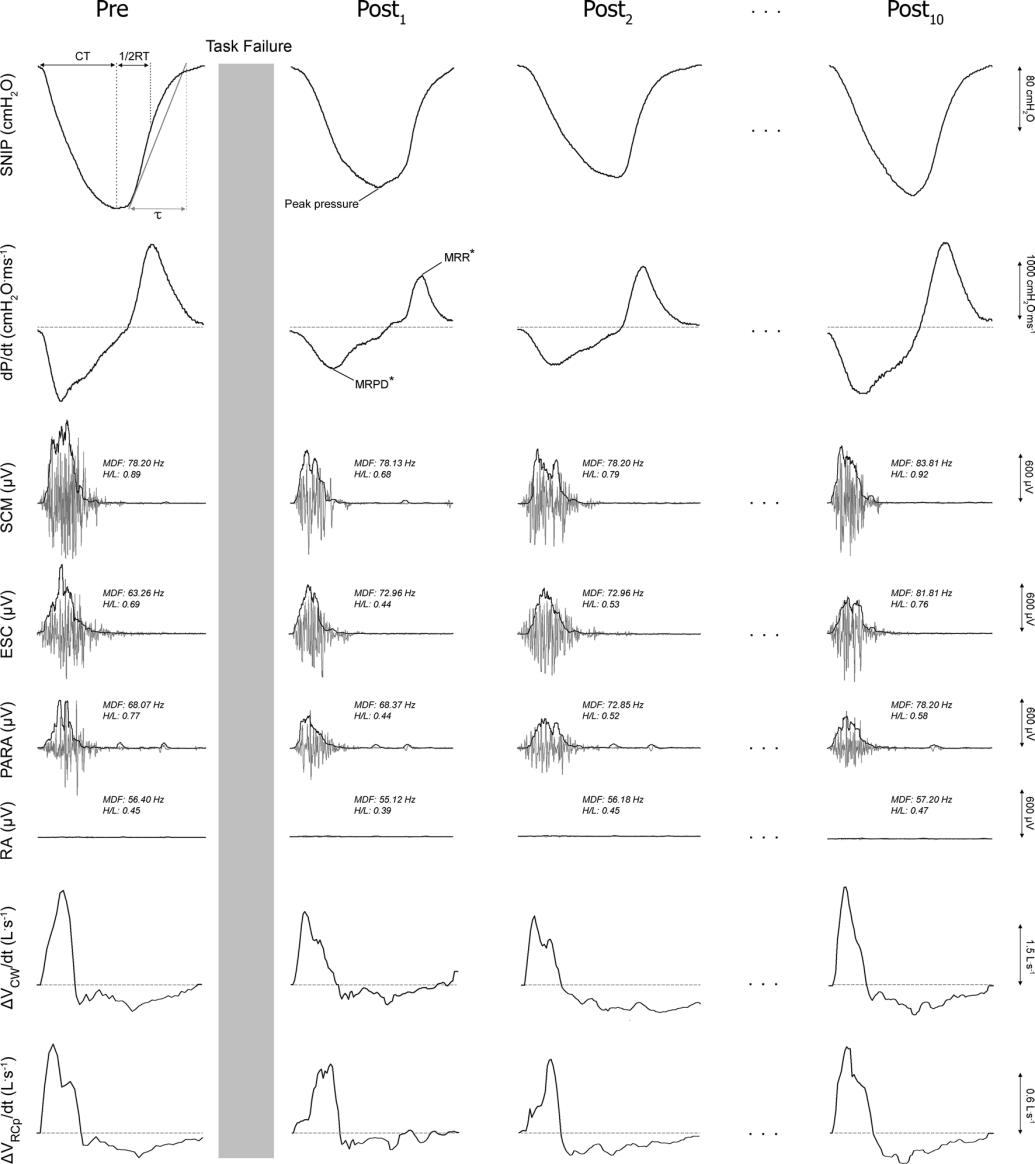
Representative tracings of the sniff pressure curve and its derivative (dP/dt), surface electromyography of sternocleidomastoid (SCM), scalene (ESC), parasternal (PARA) and rectus abdominis (RA) muscles, and shortening velocity index of the global (ΔV_CW_/dt) and ribcage (ΔV_RCp_/dt) inspiratory muscles obtained in one subject before (Pre) and after (Post_1_, Post_2_ … Post_10_ – recovery) the fatigue protocol. Grey and black traces of the surface electromyography signal represent raw and root mean squared data, respectively. The calculation of the time constant of the relaxation curve (τ) is shown by drawing a tangent line at the initial rate of decay. Median frequency (MDF) and high/low frequency power (H/L) are also expressed. SNIP: sniff nasal inspiratory pressure; CT: contraction time; ½RT: half-time of relaxation curve; MRPD: maximum rate of pressure development; MRR: maximum relaxation rate; µV: microvolts; cmH_2_O: centimeters of water; L·s: liter per second. *MRPD and MRR are represented in the figure without normalization by peak pressure.

Here, as in the pre-fatigue phase, sEMG signals were captured synchronously with SNIP maneuvers and the OEP system (Fig. 1). For each sEMG parameter, values were expressed relative to pre-fatigue values.

### Statistical analysis

Data are presented as mean ± SD, unless otherwise stated. Data normality was verified using Shapiro–Wilk test. One-way ANOVA for repeated measures or Friedman’s test was applied to compare SNIP, OEP, mechanical power, RMS, H, L, H/L ratio, and median frequency between pre-fatigue and recovery moments. To avoid type I error due to the multiplicity of post-fatigue moments, the two-stage false discovery rate test correction (using a threshold value of 5%) was applied in the event of statistical significance instead of Bonferroni’s or Dunn’s post hoc test^46^. For changes in end-expiratory chest wall volumes and coefficients of variation of all SNIP variables within maneuvers, see Supplementary information.

Regression analysis was applied in the median frequency and H/L variables to ascertain whether inspiratory muscles were developing fatigue during the fatigue protocol, and the regression curve that fitted maximum values in a least-square sense was used as fatigue index. Coefficients of determination (r^2^), slopes, and time constants were calculated for all regression analyses during fatigue and recovery moments. For regression analysis during recovery, the starting point at time zero corresponded to the last point of the fatigue protocol in each muscle. For linear regressions, time constants were calculated as the inverse value of the slope of the regression line^7^, whereas for non-linear regressions, slopes were calculated as the derivative of the exponential equation at the beginning of the fatigue protocol (Supplementary information 2). As median frequency and H/L may present different decay patterns^47,48^, muscle fatigue development was confirmed if the following criteria were achieved: (1) negative slope, in case of linear regressions^47^; (2) decrease to levels below 60% of values recorded at the beginning of the fatigue protocol^49^, in case of exponential regressions. For RMS analyses during the fatigue protocol, see Supplementary information.

Regression analysis was also applied to study recovery behavior (if linear or exponential) of median and H/L frequencies, SNIP parameters, shortening velocity indexes, and mechanical power data. Additionally, a correlation matrix was applied to study relationships between relative changes of MDF_rcm_ (median frequency), H/L_rcm_, H_rcm_, L_rcm_, and relative changes of contraction and relaxation parameters, mechanical power, and shortening velocity data during recovery. For these analyses, r^2^ and p-values were computed.

Inferential data analysis was performed using GraphPad Prism⁠ software version 8.01 for Windows. *P*-value of < 0.05 (2-sided) was considered statistically significant.

## Results

### Individuals

Twenty-six self-reported healthy adults (16 males and 10 females) were included. Anthropometric, spirometric, and respiratory muscle strength data are shown in Table 1.

**Table 1:** Subjects characteristics.

	Healthy
Subjects _(n)_	26 (16 M : 10F)
Age _(years)_	25 ± 3
Height _(m)_	1.70 ± 0.07
Weight _(kg)_	64.14 ± 11.12
BMI _(kg/m_^2^_)_	23.01 ± 2.77
FVC _(L)_	4.46 ± 0.66
FVC _%pred_	93.12 ± 7.21
FEV_1 (L)_	3.68 ± 0.48
FEV_1%pred_	91.40 ± 6.12
FEV_1_/FVC	0.82 ± 0.05
FEV_1_/FVC _%pred_	98.27 ± 6.40
FEF_25-75%(L/s)_	3.75 ± 0.71
PEF _(L/s)_	8.01 ± 1.83
MIP _(cmH2O)_	117.40 ± 8.59
MIP _%pred_	98.40 ± 6.63
MEP _(cmH2O)_	128.80 ± 28.67
MEP _%pred_	102.17 ± 17.14
SNIP _(cmH2O)_	106.50 ± 22.77
SNIP _(%pred)_	95.50 ± 11.40

FVC: forced vital capacity; FEV_1_: forced expired volume in 1 s; FEF_25-75%_: forced expiratory flow at 25–75% of FVC; PEF: peak expiratory flow; MIP: maximal inspiratory pressure; MEP: maximal expiratory pressure; SNIP: Sniff nasal inspiratory pressure; m: meters, kg: kilograms; L: liters; %pred: percentage of predicted; cmH_2_O: centimeters of water; M male; F: female. Values are shown as mean ± SD.

### sEMG power spectrum during fatigue protocol and recovery

#### Fatigue protocol

**Figure 2:**
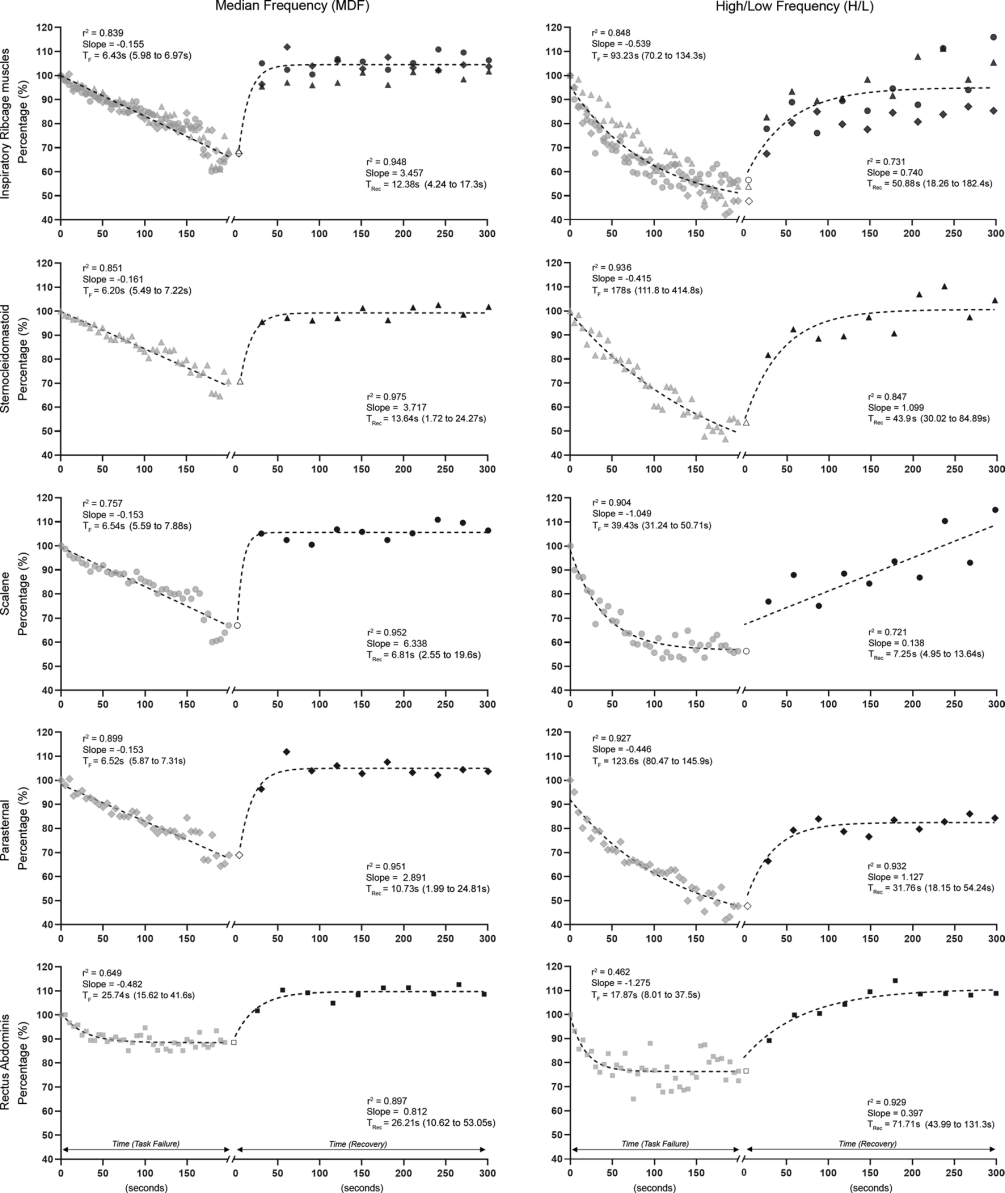
Time courses of normalized median frequency (left panels) and high/low ratio (right panels) of inspiratory ribcage muscles (mean values of the three inspiratory muscles studied), sternocleidomastoid (triangles), scalene (circles), parasternal (diamonds), and rectus abdominis (squares) muscles, respectively, during fatigue and recovery moments. Each point during the fatigue protocol (grey symbols) represents data averaged from 5 s, while during recovery (black symbols), each point represents data extracted from each SNIP maneuver (i.e., ten SNIP maneuvers with an interval of 30 s in between). All variables are normalized to their initial value during the fatigue protocol. In each muscle, the starting point at time zero (white symbols) corresponds to the last point of the fatigue protocol. Lines represent regression curves that fitted maximum values in a least-square sense, and equations are shown in Supplementary information 5. T_F_ and T_Rec_ represent the time constant during fatigue protocol and recovery, respectively, and numbers in parentheses are the 95% confidence interval. %: percentage; s: seconds.

Overall, a mean inspiratory load of − 82.4 ± 10 cmH_2_O and mean T_lim_ of 200 ± 50 s fatigued inspiratory ribcage muscles, as confirmed by MDF_rcm_ and H/L_rcm_ parameters (Fig. 2). Median frequency (Fig. 2, left panels) decreased linearly during the fatigue protocol in all muscles, with SCM presenting the highest slope of decay (slope = ‒0.161, r^2^ = 0.851). H/L (Fig. 2, right panels) decreased early before mechanical fatigue (i.e., before reaching T_lim_) and exponentially with time, mainly due to ESC and PARA muscles. In particular, the reduction in high-frequency band was more pronounced (i.e., higher slopes) than the increase in low-frequency, especially for ESC muscle (slope = ‒0.895, r^2^ = 0.921) (Supplementary information 3).

H/L values for RA decreased ~ 22% with a plateau at 50 s (Fig. 2, last row), indicating no fatigue development, despite negative median frequency slope.

For inspiratory muscles, fatigue time constants of median frequency were very close and ranged between 6.20 s in the SCM and 6.54 s in the ESC muscle. Conversely, H/L was characterized by higher time constants, particularly the SCM muscle (178 s). SCM was also the most activated muscle during the fatigue protocol (~ 50% increase of RMS initial value), with %RMS values higher than ESC, PARA, and RA muscles (*p* < 0.0001) during the first 100–200 s (Supplementary information 4).

#### Recovery phase

MDF_rcm_ increased exponentially within the first 30 s after fatigue, with a mean time constant of 12.38 s, mainly due to the slow SCM recovery. H/L_rcm_ also followed an exponential recovery pattern with a slow time constant (50.88 s) due to SCM muscle. Recovery of high-frequency was slower than low-frequency values in the SCM and ESC muscles (Supplementary information 3).

Only %RMS change of the SCM remained significantly lower than pre-fatigue values (*p* < 0.0001), with no significant changes in %RMS_rcm_. H/L_rcm_ was significantly lower (*p* < 0.001) until the 7^th^ maneuver after fatigue, mainly due to H/L of the PARA muscle (*p* < 0.001). No differences were observed in median frequency values of all muscles studied or H/L of SCM, ESC, and RA muscles (data not shown).

Regression equations that fitted all power spectrum parameters during fatigue development and recovery are shown in the Supplementary information 5.

### Sniff curve parameters during recovery

A linear relationship was observed between MRPD and peak pressure (r = − 0.844, r^2^ = 0.713, *p* < 0.0001), justifying MRPD normalization to peak pressure.

**Figure 3:**
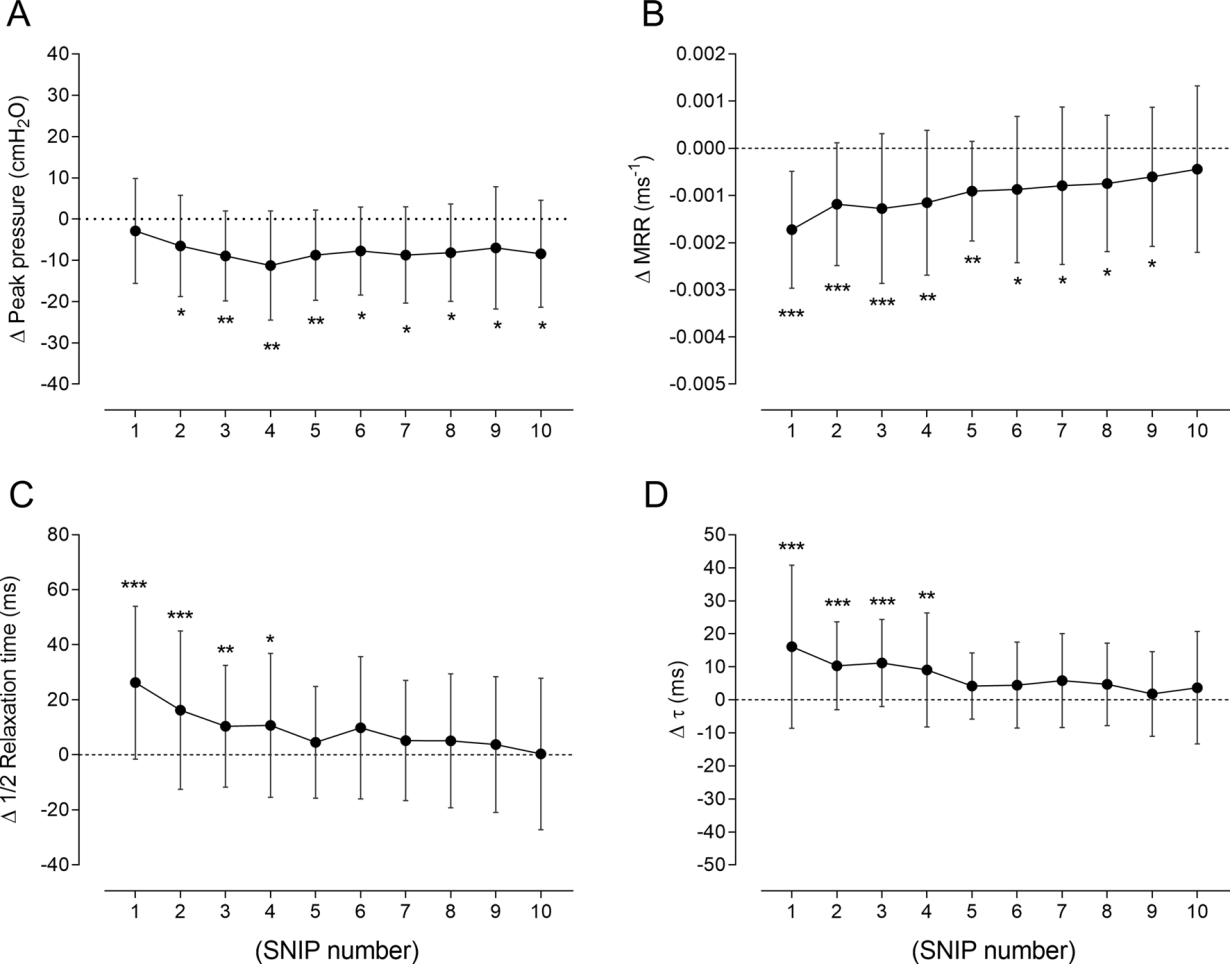
Data are shown as mean ± SD of all 26 subjects included in the study. Changes in peak pressure (panel **A**), maximum relaxation rate (MRR—panel **B**), half-time of relaxation curve (½RT—panel **C**), and time constant of the relaxation curve (τ—panel **D**) obtained from sniff curves of all subjects during recovery from fatigue protocol were compared with pre-fatigue values (line at zero). cmH_2_O: centimeters of water; ms: milliseconds. *p < 0.05; **p < 0.01; and ***p < 0.0001.

During recovery, changes in peak SNIP pressure (Fig. 3A) were significantly lower than pre-fatigue values between the 2nd and 10th maneuvers (*p* < 0.0001), with no differences in contractile parameters. Conversely, all relaxation parameters changed significantly after fatigue (all *p* < 0.0001). MRR (Fig. 3B) fell by 18.3% and recovered to pre-fatigue values in the 10th maneuver, while ½RT (Fig. 3C) and τ (Fig. 3D) rose 21.8% and 40.8%, respectively, in the 1st maneuver post-fatigue and returned to baseline values in the 5th SNIP maneuver (absolute values are shown in Supplementary information 6). Relaxation was significantly more influenced by fatigue than contractile properties until the 5th maneuver (*p* = 0.01), according to contraction-relaxation coupling.

### OEP measures during recovery

#### Chest wall and compartmental volumes

**Figure 4:**
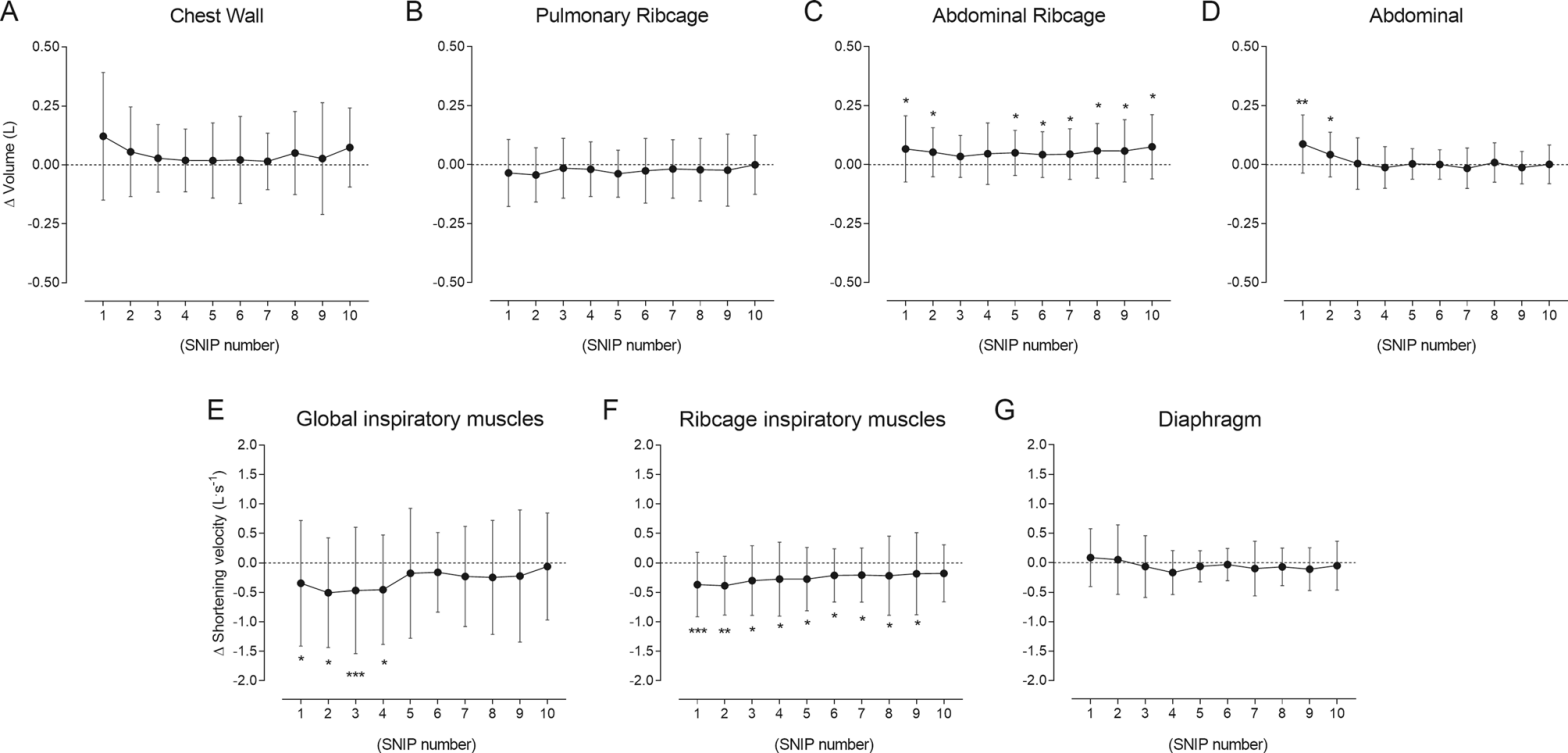
Data are shown as mean ± SD of all 26 subjects included in the study. Changes in chest wall (panel **A**) and compartmental [pulmonary ribcage (panel **B**), abdominal ribcage (panel **C**), and abdominal (panel **D**)] volumes, shortening velocity index of the global inspiratory (ΔV_CW_/Ti– panel **E**), ribcage (ΔV_RCp_/Ti – panel **F**), and diaphragm (ΔV_AB_/Ti – panel **G**) muscles obtained during the SNIP maneuvers during recovery were compared with pre-fatigue values (line at zero). *p < 0.05; **p < 0.01; and ***p < 0.0001.

Figure 4 reports changes in OEP values during recovery (absolute values are shown in Supplementary information 7). Only ΔV_RCa_ (*p* = 0.01) and ΔV_AB_ (*p* = 0.01) changed significantly after fatigue. ΔV_CW_/Ti was significantly lower than pre-fatigue values until the 4^th^ maneuver (*p* = 0.02), while ΔV_RCp_/Ti was lower between the 1^st^ and 9^th^ maneuvers (*p* = 0.01). End-expiratory chest wall volumes were not different between recovery and pre-fatigue phases (Supplementary information 8).

#### Mechanical power

**Figure 5:**
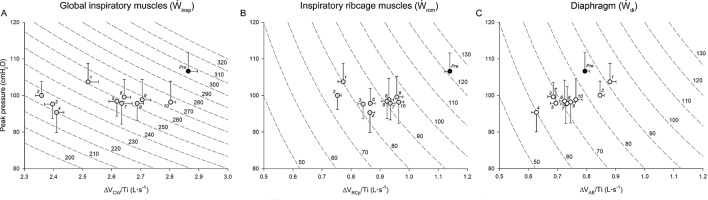
Relationships between mean values of peak pressure (*y*-axis) and shortening velocity index (*x*-axis) of global inspiratory (ΔV_CW_/Ti – panel **A**), ribcage (ΔV_RCp_/Ti –panel **B**), and diaphragm (ΔV_AB_/Ti – panel **C**) muscles. Dotted lines represent isopleths of different levels of mechanical power (Ẇ) from 200 to 320 cmH_2_O·L·s^-1^ (left panel), and from 50 to 130 cmH_2_O·L·s^-1^ (middle and right panels), according to the relationship Ẇ = pressure × shortening velocity. Black dots represent mean pre-fatigue values of all 26 subjects included. The remaining dots represent mean peak pressure values obtained from SNIPs performed during recovery (significant values [grey] and non-significant values [white]). Vertical and horizontal bars represent ± SE values of peak pressure and shortening velocity indexes, respectively. Numbers near dots represent the order of SNIP maneuvers during recovery.

Figure 5 shows that fatigue evoked significant changes in mechanical power. Ẇ_insp_ decreased significantly (*p* = 0.005), mainly due to Ẇ_rcm_ (*p* = 0.03), and returned to pre-fatigue values in the 10^th^ maneuver. Immediately after fatigue, most reduction of Ẇ_insp_ and Ẇ_rcm_ was rather due to shortening velocity (16.34 and 32.15%, respectively) than pressure (2.69%). Even in the lowest Ẇ_insp_ and Ẇ_rcm_ values (4^th^ and 2^nd^ maneuvers, respectively), shortening velocity played a major role (15.82% and 32.15%, respectively) than pressure (10.56% and 6.10%, respectively) (Supplementary information 9).

### Relationships during recovery

All r^2^ and p-values of the correlation matrix are present in Table 2A and B, respectively. Relaxation rates (MRR, τ, and ½RT) associated significantly with ΔV_CW_/Ti, ΔV_RCp_/Ti, H/L_rcm_, H_rcm_, L_rcm_, Ẇ_rcm_, and MDF_rcm_ changes.

**Table 2:** R^2^ (A) and *p*-values (B) of the correlation matrix between the contractile and relaxation parameters, shortening velocity indexes, mechanical power and surface electromyographic changes of inspiratory ribcage muscles during recovery from fatigue.

(A)																			
	Pressure	TPS	CT	MRDP	MRPD/Peak	MRPD/MRR	MRR	τ	½RT	MDF_rcm_	H/L_rcm_	H_rcm_	L_rcm_	ΔV_CW_/Ti	ΔV_RCp_/Ti	ΔV_AB_/Ti	Ẇ_insp_	Ẇ_rcm_	Ẇ_di_
Pressure	-	0.763	0.051	**0.021**	0.981	0.873	0.099	0.081	0.098	0.201	0.206	0.490	0.141	0.746	0.107	** < 0.001**	0.743	0.342	< 0.001*
TPS		–	0.935	0.628	0.630	0.931	0.641	0.579	0.605	0.898	0.691	0.461	0.308	0.234	0.962	0.504	0.232	0.945	0.637
CT			–	**0.001**	0.180	0.454	0.326	0.178	0.132	0.580	0.681	0.900	0.767	0.723	0.267	0.064	0.939	0.541	0.051
MRDP				–	**0.023**	0.880	**0.038**	**0.022**	**0.008**	0.159	0.101	0.158	0.154	0.175	0.055	0.066	0.393	0.132	0.085
MRPD/Peak					–	0.973	0.283	0.227	0.236	0.482	0.292	0.172	0.543	0.121	0.373	0.891	0.121	0.324	0.737
MRPD/MRR						–	**0.011**	0.034	0.087	**0.014**	0.07	**0.007**	0.087	0.135	0.052	0.695	0.135	**0.013**	0.629
MRR							–	** < 0.001**	** < 0.001**	**0.007**	** < 0.001**	**0.002**	**0.012**	**0.017**	** < 0.001**	0.089	0.064	** < 0.001**	0.138
τ								–	** < 0.001**	**0.002**	**0.006**	**0.006**	**0.023**	**0.045**	**0.004**	0.067	0.100	**0.006**	0.115
½RT									–	**0.019**	**0.003**	**0.011**	**0.003**	**0.067**	**0.004**	0.068	0.197	**0.009**	0.057
MDF_rcm_										–	**0.003**	**0.002**	**0.018**	0.308	0.074	0.153	0.412	0.073	0.123
H/L_rcm_											–	** < 0.001**	**0.002**	0.06	0.06	0.567	0.081	0.07	0.538
H_rcm_												–	**0.004**	0.369	0.904	0.265	0.226	0.608	0.125
L_rcm_													–	0.175	0.099	0.279	0.342	0.122	0.175
V_CW_/Ti														–	**0.009**	0.696	< 0.001*	0.003*	0.779
V_Rcp_/Ti															–	0.044	0.054	< 0.001*	0.199
V_AB_/Ti																–	0.863	0.156	< 0.001*
Ẇ_insp_																	–	**0.015**	0.394
Ẇ_rcm_																		–	0.413
Ẇ_di_																			–

(B)																			
	TPS	CT	MRDP	MRPD/Peak	MRPD/MRR	MRR	τ	½RT	MDFrcm	H/Lrcm	Hrcm	Lrcm	ΔVCW/Ti	ΔVRCp/Ti	ΔVAB/Ti	Ẇinsp	Ẇrcm		
Pressure	-	0.012	0.574	**0.518**	0.001	0.003	0.302	0.331	0.689	0.195	0.190	0.061	0.250	0.013	0.291	**0.801**	0.014		
TPS		–	0.001	0.030	0.030	0.001	0.028	0.040	0.187	0.002	0.020	0.069	0.128	0.171	0.001	0.057	0.172		
CT			–	**0.757**	0.212	0.071	0.120	0.214	0.510	0.040	0.022	0.002	0.011	0.016	0.151	0.550	0.001		
MRDP				–	**0.494**	0.003	**0.437**	**0.503**	**0.779**	0.232	0.300	0.232	0.236	0.216	0.386	0.480	0.092		
MRPD/Peak					–	0.000	0.142	0.176	0.413	0.063	0.137	0.219	0.047	0.273	0.100	0.002	0.273		
MRPD/MRR						–	**0.577**	0.448	0.322	**0.449**	0.570	**0.619**	0.321	0.257	0.395	0.020	0.257		
MRR							–	**0.930**	**0.870**	**0.516**	**0.811**	**0.734**	**0.565**	**0.533**	**0.823**	0.318	0.364		
τ								–	**0.830**	**0.465**	**0.627**	**0.627**	**0.497**	**0.412**	**0.657**	0.358	0.301		
½RT									–	**0.418**	**0.697**	**0.673**	**0.670**	**0.360**	**0.673**	0.436	0.198		
MDF_rcm_										–	**0.677**	**0.714**	**0.525**	0.128	0.344	0.237	0.085		
H/L_rcm_											–	**0.912**	**0.725**	0.410	0.430	0.042	0.332		
H_rcm_												–	**0.656**	0.101	0.002	0.152	0.177		
L_rcm_													–	0.216	0.303	0.143	0.112		
V_CW_/Ti														–	**0.600**	0.020	0.925*		
V_Rcp_/Ti															–	0.414	0.388		
V_AB_/Ti																–	0.003		
Ẇ_insp_																	–		
Ẇ_rcm_																			
Ẇ_di_																			

(A) Significant r^2^ values are bolded. TPS: time to peak shortening; CT: contraction time; MRPD: maximum rate of pressure development; MRPD/Peak: maximum rate of pressure development normalized by peak pressure; MRR: maximum relaxation rate; τ : time constant of pressure decay (tau); ½RT: half-time of the relaxation curve; MDF_rcm_: median frequency of the inspiratory ribcage muscles; H/L_rcm_: high/low ratio of the inspiratory ribcage muscles; H_rcm_: high-frequency band of the inspiratory ribcage muscles; L_rcm_: low-frequency band of the inspiratory ribcage muscles; ΔV_CW_/Ti: shortening velocity index of global inspiratory muscles; ΔV_RCp_/Ti: shortening velocity index of inspiratory ribcage muscles; ΔV_AB_/Ti: shortening velocity index of the diaphragm; Ẇ_insp_: mechanical power of global inspiratory muscles; Ẇ_rcm_: mechanical power of inspiratory ribcage muscles; Ẇ_di_: mechanical power of diaphragm muscle. *Mechanical power is the product of peak pressure and shortening velocity indexes.

(B) Significant *p*-values are bolded. TPS: time to peak shortening; CT: contraction time; MRPD: maximum rate of pressure development; MRPD/Peak: maximum rate of pressure development normalized by peak pressure; MRR: maximum relaxation rate; τ : time constant of pressure decay (tau); ½RT: half-time of the relaxation curve; MDF_rcm_: median frequency of the inspiratory ribcage muscles; H/L_rcm_: high/low ratio of the inspiratory ribcage muscles; H_rcm_: high-frequency band of the inspiratory ribcage muscles; L_rcm_: low-frequency band of the inspiratory ribcage muscles; ΔV_CW_/Ti: shortening velocity index of global inspiratory muscles; ΔV_RCp_/Ti: shortening velocity index of inspiratory ribcage muscles; ΔV_AB_/Ti: shortening velocity index of the diaphragm; Ẇ_insp_: mechanical power of global inspiratory muscles; Ẇ_rcm_: mechanical power of inspiratory ribcage muscles; Ẇ_di_: mechanical power of diaphragm muscle. *Mechanical power is the product of peak pressure and shortening velocity indexes.

### Patterns of recovery

**Figure 6:**
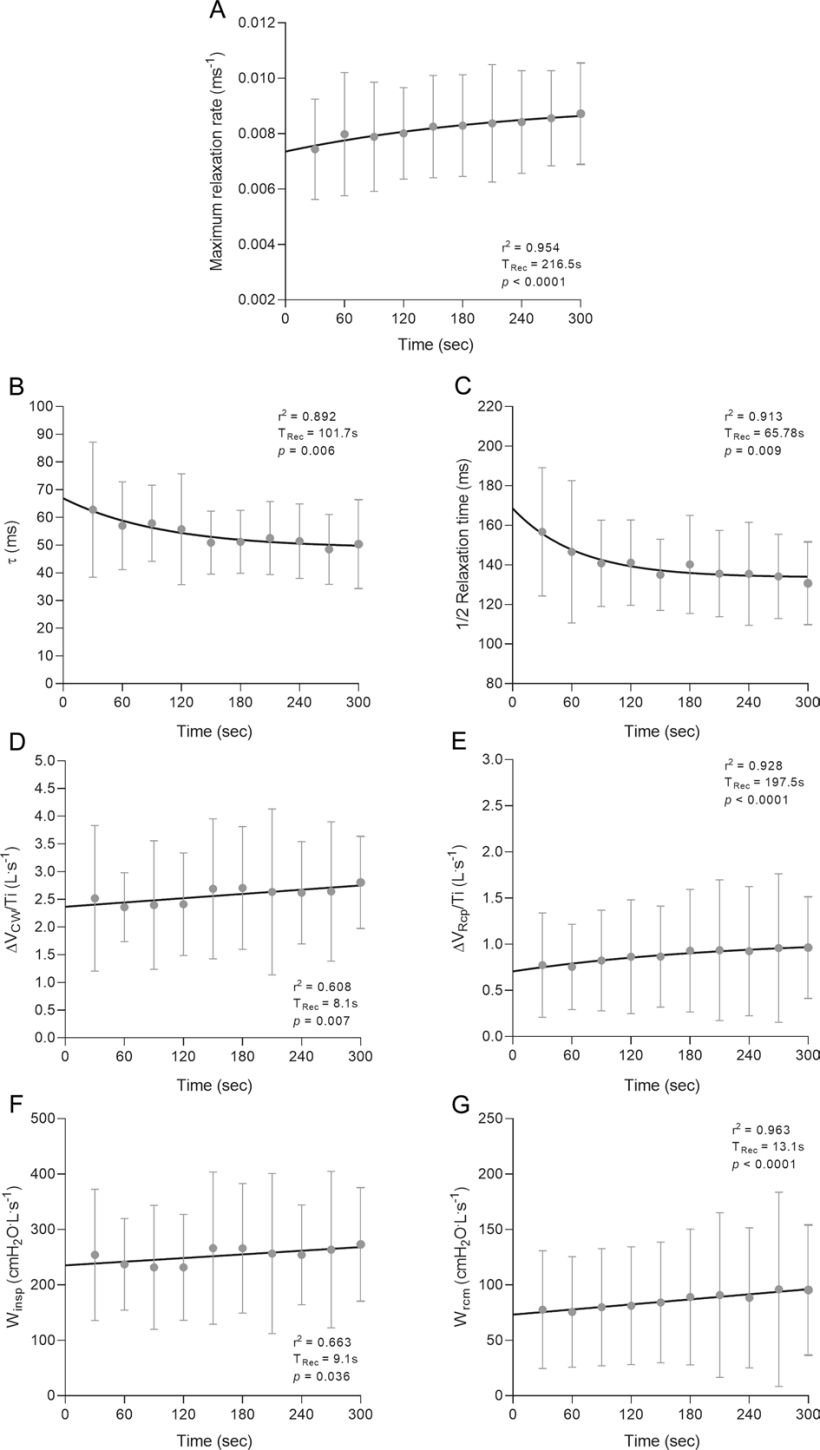
Time course of the recovery pattern of maximum relaxation rate (panel **A**), time constant of the relaxation curve (τ—panel **B**), half-time of relaxation curve (½RT—panel **C**), mechanical power of global inspiratory (Ẇ_insp_—panel **D**) and inspiratory ribcage muscles (Ẇ_rcm_—panel **E**), and shortening velocity indexes of global inspiratory (ΔV_CW_/Ti—panel **F**) and inspiratory ribcage muscles (ΔV_RCp_/Ti—panel **G**). Each point represents mean ± SD data obtained from SNIP maneuvers performed during recovery in all 26 subjects. Lines represent regression curves that fitted maximum values in a least-square sense. sec: seconds.

MRR showed the slowest recovery, followed by τ and ½RT (Fig. 6A–C). ΔV_RCp_/Ti recovered exponentially, while a straight-line relationship best described ΔV_CW_/Ti, Ẇ_insp_, and Ẇ_rcm_ recoveries (Fig. 6D–G).

#### Relationships between relaxation parameters, and ΔV_RCp_/Ti and Ẇ_rcm_ during recovery

**Figure 7:**
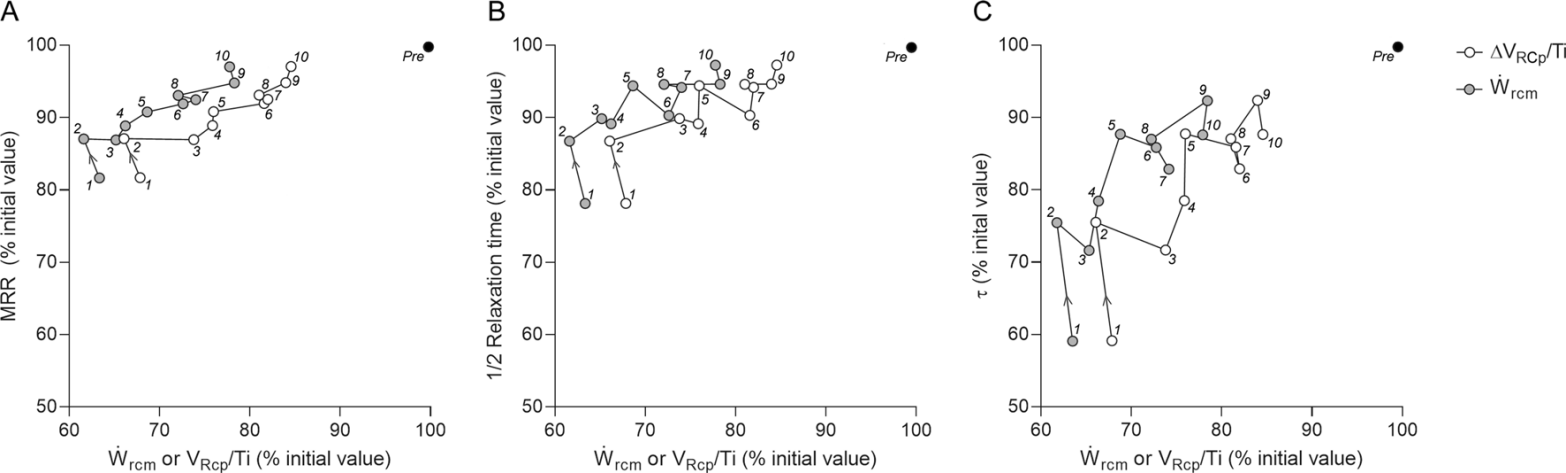
Relative mean changes of maximum relaxation rate (MRR – panel **A**), half-relaxation time (½RT – panel **B**), and time constant of the relaxation curve (τ – panel **C**) in relation to mean changes in shortening velocity index (ΔV_RCp_/Ti – white dots) and mechanical power (Ẇ_rcm_ – grey dots) of the inspiratory ribcage muscles during recovery in all 26 subjects included. Numbers near dots represent the order of the SNIP maneuvers during recovery. Note that pre-fatigue values are 100% for y- and x-axis (black dots) and relaxation parameters returned to values above 90% of initial values, while both ΔV_RCp_/Ti and Ẇ_rcm_ remain below 90%.

Figure 7A–C shows the relationships between MRR, ½RT, and τ relative mean changes as a function of relative mean change in ΔV_RCp_/Ti or Ẇ_rcm_ during recovery. Relaxation rates were characterized by rapid recovery immediately after the fatigue protocol, and only MRR and ½RT returned to mean values close to pre-fatigue. Both ΔV_RCp_/Ti and Ẇ_rcm_ were almost mirrored with relaxation parameters; however, they did not recover completely.

## Discussion

The main findings of this study are that (1) inspiratory ribcage muscles have different responses (i.e., different underlying mechanisms) to fatigue and recovery, as reflected by changes in spectral sEMG parameters, (2) loss of mechanical power for the ribcage muscles is due to reduced shortening velocity with a long lasting reduction in pressure generation, and (3) relaxation properties are better associated with recovery from fatigue than contractile properties.

sEMG spectral analysis revealed a decrease in median frequency during fatigue in all muscles, with a faster decay in the SCM, probably due to different fiber type composition^50,51^ since SCM has a higher proportion of type II fibers (65%) than ESC and PARA muscles (39% and 38%, respectively)^52,53^. This result is not surprising since recruitment of this fiber type is preferential at higher loads and more prone to fatigue^54^.

Apart from similar fiber type distribution, H/L fatigue time constant of PARA was threefold greater than ESC muscle, which may be explained by the amount of inspiratory drive received and mechanical advantage during loaded breathing. Inspiratory muscles in the neck are recruited according to their mechanical advantage, and the inspiratory effect of ESC and SCM together is double the effect of parasternals^55^, implicating greatest inspiratory drive, higher motor unit firing rates^56^, and faster fatigue. Furthermore, inspiratory load imposition may alter timing and magnitude of motor discharges to the PARA, inhibiting its activity^57^ during fatigue phase and leading to a longer time constant.

The pattern of change of high- and low-frequency corroborates with Kadefors et al.^8^, suggesting that changes in both bands do not occur synchronously and are probably of different origins. Shifts in power spectrum are also consistent with decreased conduction velocity and elongation of myopotential waveforms^48,58^. Concerning RA, the decreased H/L was probably the result of muscle co-activation during inspiratory loaded breathing^59^. Activation of RA during loaded breathing occurs to reduce end-expiratory lung volume (i.e., functional residual capacity), improve length for force development of diaphragm, and store elastic energy in abdominal and thoracic walls for subsequent inspiration^60,61^. However, the absence of RA fatigue in our study suggests no active recruitment during expiration^59^ and confirms the maintenance of functional residual capacity throughout the fatigue phase.

The SNIP maneuver also evoked contraction of other inspiratory ribcage muscles^14^. In this context, and along with the fact that sniffs were accompanied by true abdominal relaxation and stress adaptation of the lungs and chest wall does not modify pressure decay^62^, it can be confirmed that relaxation portion of the curve represents the rate of relaxation of inspiratory ribcage muscles.

Regarding contraction properties, fatigue only induced a decrease in peak pressure generation. In particular, MRPD/Peak did not change, indicating no disproportionate effect of fatigue on maximal capacity of pressure development for peak pressure production. Thus, the reduction in the non-normalized MRPD resulted primarily from a decline in peak pressure, which may be caused by fatigue-induced deleterious alterations in the contractile apparatus of muscle fibers^26^. In this sense, the increased contraction-relaxation coupling was mainly due to decreases in MRR, while alterations in the contractile apparatus led to peak pressure changes rather than MRPD.

Fatigue can also contribute to slowing of relaxation by affecting different cellular mechanisms^10^. Relative changes in relaxation properties were similar to those found for human diaphragm^63^ and was probably caused by different metabolic factors^64^. Even though relationships between MRR and τ were present during recovery, both τ and ½RT recovered faster than MRR. Moreover, the lack of relationship between relaxation rates and peak pressure indicates that recovery of these properties may take place by different mechanisms. Although relaxation kinetics and associations with MRR, τ, and ½RT were not studied here, the fatigue protocol may have affected both the slow and fast phases of the relaxation curve differently since they are determined by distinct biochemical pathways or different and/or coincident mechanisms during cross-bridge detachment^65,66^.

Within the limits of this study, diaphragmatic influence on peak pressure generation cannot be ruled out since V_RCa_ and V_AB_ (diaphragmatic action)^12^ increased during recovery, which may have influenced pressure generation immediately after the fatigue protocol. Associations between peak pressure and V_AB_/Ti also suggest diaphragmatic contribution to peak pressure generation to counteract the loss of Ẇ_rcm_ and maintain Ẇ_insp_. Although pressure generation is drastically reduced during selective ribcage contraction^55^, T_lim_ values were close to other studies^67^ and lower than expected to diaphragm fatigue when controlling duty cycle between 0.2 and 1^48^, indicating selective overload and earlier ribcage muscle fatigue in the face of a specific recruitment pattern. Nevertheless, the contribution of different ribcage muscles and/or alternating recruitment to sustain the imposed load cannot be excluded^7,68^.

Loss of power during fatigue results from different combinations of changes in force, slowing of shortening velocity, or both. Westerbland et al.^69^ observed that force generation was reduced when calcium release was inhibited from the sarcoplasmic reticulum, with no shortening velocity effects. Conversely, Piazzesi et al.^70^ showed that shortening velocity was dependent on attachment rates between myosin and actin. In this sense, if slowing of relaxation is a function of changes in the contractile apparatus, then changes in force–velocity characteristics would reduce force and power output for a given shortening velocity^71,72^.

In the present study, loss of Ẇ_insp_ after fatigue was due to both reductions in force and velocity. Also, ΔV_RCp_/Ti and Ẇ_rcm_ were associated with MRR, τ, and ½RT during recovery, and recovery time constant of both the ΔV_RCp_/Ti and MRR were relatively close, indicating that ΔV_RCp_/Ti changes are probably a good indicator of MRR changes in vivo. These are consistent with literature from limb muscles in humans, in which force and velocity follow different recovery profiles^73–75^ with fatigue-related changes in shortening velocity closely related to changes in contractile slowing^17,73^. Furthermore, strong temporal relationships and time courses indicate that relaxation rates may recover faster than shortening velocity or mechanical power; however, the long-lasting depression in peak pressure (i.e., force) during recovery may have contributed to incomplete recovery of power, especially in the inspiratory ribcage muscles. This fits previous findings that the primary cause of fatigue in human intercostal muscles is decreased force caused by impaired calcium release with no indications of contractile slowing being the major cause of ribcage muscle fatigue^18^. Therefore, these results suggest that (1) different inspiratory muscles may have different underlying mechanisms of fatigue, with some muscles more susceptible to contractile slowing and others more susceptible to decreased force, and (2) MRR, τ, and ½RT may share common underlying mechanisms with shortening velocity during recovery from acutely fatigued inspiratory ribcage muscles, supporting Jones et al.^17^.

Regarding %RMS, only %RMS change of SCM remained significantly lower than pre-fatigue values, with no significant changes in %RMS_rcm_. According to Stegeman and Linssen^76^, %RMS is strongly dependent on electrophysiological changes within the muscle, and its decline after fatiguing tasks is accompanied by decreases in electrical excitation reaching the muscle^77^. As SCM was the most activated during fatigue phase, reflex inhibition of voluntary muscle activation may have occurred during recovery due to reduced central motor drive to limit the risk of further harm^78^. Nevertheless, the unchanged %RMS_rcm_ and decreased peak pressure during recovery indicates that inspiratory ribcage muscle excitation was not altered as a whole, and changes observed in relaxation parameters, shortening velocity indexes, and mechanical power were probably due to changes in the contractile system of the studied muscles^79^.

Recovery time constant of median frequency was lower than observed in relaxation rates or H/L, indicating differences in how these parameters are coupled to metabolic changes during recovery. Indeed, restitution of mechanical properties and relaxation rates need a more extended rest bout than median frequency^80^. To our knowledge, literature lacks data regarding median frequency recovery after dynamic inspiratory ribcage muscle fatigue and presents conflicting data regarding time constants during recovery since it is dependent on muscle type and fatigue protocol^81,82^. Despite this, muscle recovery is dependent on aerobic metabolism, which would explain why PARA and ESC muscles presented higher time constants than SCM^83^.

Even though different biochemical mechanisms determine relaxation rates and H/L parameters, changes in MRR and τ might allow predicting fatigue since temporal relationships with H/L are present during a fatigue protocol^84^. We observed for the first time that recovery of both H/L_rcm_ and MDF_rcm_ were independent of peak pressure and proportional to recovery of relaxation rates, suggesting that 1) relaxation parameters are useful predictors of inspiratory ribcage muscle recovery, and 2) force generation and sEMG power spectrum of ribcage muscles recover at different rates. Also, changes in H/L_rcm_ were progressively reversible and H/L_rcm_ time constant (50.88 s) was within the range reported for the diaphragm during recovery (35 to 55 s, mean time constant of 42 s)^48^. In this sense, ribcage muscles may present electromyographic patterns similar to diaphragm during recovery, and H/L_rcm_ can be considered an index of inspiratory ribcage muscle recovery.

Interestingly, H/L restitution in the PARA muscle was not completed after 5 min of rest, probably due to incomplete restoration of muscle circulation and altered conduction velocity^85^. Changes in blood flow reflect changes in metabolic demands and muscle contraction, and normal conduction velocity is dependent on blood flow for metabolic by-product removal. Unlike the ESC^45^, blood flow distribution of PARA (2^nd^ intercostal space) is not increased during breathing against resistive loads compared with quiet breathing^86^. Thus, we believe that, together with relative mechanical disadvantage^55^, low perfusion to PARA led to a slower removal of by-products and slower H/L restitution.

Decay in the power content of high-frequency band reflects progressive failure of action potential generation, while its increase during recovery indicates reparative mechanisms^87^. If true, recovery of this parameter, rather than low-frequency band, better accompanied recovery of relaxation parameters. However, the inhomogeneous restitution of the high-frequency band in all studied muscles led us to believe that other factors occur during recovery from fatigue^9,85^.

### Strengths and limitations

This study provides the first detailed description of contraction and relaxation parameters, shortening velocity, mechanical power, and power spectrum sEMG changes during fatigue and recovery of three inspiratory ribcage muscles during resistive dynamic voluntary contractions. All measurements were obtained non-invasively and can be potentially applied to a wide range of patients and conditions.

SNIP was chosen instead of maximal inspiratory pressure to assess inspiratory ribcage muscles because it is fast and easy to perform and allows a more relaxed expiration. Regarding the lack of imposed breathing pattern and flow rate control, we are aware that chest wall distortion, thoracic gas compression, and muscle fiber shortening may vary during the fatigue phase and influence metabolic requirements of muscle fibers^88^. Also, due to volitional nature of SNIP maneuvers, effort and/or motivation could potentially modify results. Nonetheless, the fatiguing protocol was performed spontaneously to maintain the characteristic use of inspiratory threshold loads.

Indirect estimation of velocity of shortening using OEP can also be considered a limitation of the study. However, instantaneous diaphragm length variations can be estimated from ΔV_AB_^12^ when considering the three compartmental model of the chest wall^89^. Also, dVrca/dVab ratio during pre and post moments was constant (Supplementary information 10), allowing shortening velocity estimation. The absence of sEMG measurement of external intercostals must also be highlighted since it has a greater inspiratory effect and mechanical advantage than PARA over the inspiratory capacity range^90^. Diaphragm sEMG was not acquired because individuals were positioned without back support (due to positioning of OEP markers) and cross-contamination with trunk muscles during postural activity could occur^91^.

During the fatigue protocol, electrocardiographic artifacts could be accounted for the one real limitation since its signal acquisition may interfere with low-frequency amplitude^92^. Although heart rate was not measured in the present study, breathing against inspiratory loads equal to or lower than 75% does not interfere with heart rate^92^. All electrodes were also positioned on the right side of the body, and a bandpass filter of 20–400 Hz was used. High-pass filtering using Butterworth filter is the most straightforward method to remove ECG artifacts, and a high-pass corner frequency between 20 and 30 Hz is best to remove ECG contamination with minimal impact on total power^93^, particularly during fatigue^94^.

Finally, apart from vocal encouragement, the inability of the central nervous system to fully recruit the involved muscles during the fatigue protocol cannot be ruled out. Thus, reduced cerebral oxygenation and motor inhibition due to central respiratory drive suppression^95,96^ may also have accounted for shortening velocity and pressure generation decrease.

### Implications and future studies

Different methods have been proposed to obtain a quantitative index of fatigue^58,97,98^. However, most studies used only one index, limiting muscle fatigue quantification (e.g., H/L is not affected in certain types of exercise producing low-frequency fatigue)^99^. In the present study, changes in median frequency and H/L of RA muscle demonstrate the need for more than one index to (1) quantify fatigue development, (2) avoid erroneous interpretations, and (3) understand possible physiological events occurring with fatigue. These must be considered, especially when studying respiratory muscle fibers in healthy and disease due to high heterogeneity and plasticity^100^.

Additionally, different adjustments of resting periods during inspiratory muscle training in healthy must be considered since (1) inspiratory ribcage muscles possess similar recovery patterns than diaphragm during fatigue and recovery, (2) relaxation properties of inspiratory muscles after fatigue can be improved with inspiratory muscle training^101^, (3) recovery of relaxation rates of ribcage muscles is relatively fast in healthy adults, and (4) no data regarding recovery patterns of relaxation parameters after inspiratory muscle training or rehabilitation are presented in the literature. Conversely, in clinical settings, for example, inspiratory muscle fatigue may impair exercise performance by altering the sensation of dyspnea or mechanical load and increasing sympathetic vasoconstrictor outflow to working muscles due to metaboreflex^102,103^. Thus, careful analyses of contractile and relaxation parameters during exercise-induced respiratory muscle fatigue and recovery are important for designing optimal strategies for patients with decreased fatigue resistance. Differences in pattern and recovery between sexes and the importance of contraction and relaxation parameters of respiratory muscles on exercise limitation must also be addressed in future studies.

## Conclusion

We have shown that a fatigue protocol using 70% of individuals’ maximal inspiratory pressure can selectively fatigue SCM, ESC, and PARA muscles in vivo by decreasing median frequency and H/L ratio. Recovery of these parameters has different time constants, with median frequency returning to pre-fatigue values faster than H/L, suggesting that different inspiratory muscles may have different underlying mechanisms of fatigue. This type of fatigue affects inspiratory ribcage muscle relaxation rates and contractile properties, leading to loss of mechanical power by decreasing shortening velocity with long-lasting reduction in pressure production. Furthermore, changes in relaxation rates, whether measured as MRR, ½RT, or τ, are strongly associated with H/L and useful predictors of inspiratory ribcage muscle recovery. Although with different time courses, relationships between shortening velocity index and relaxation rates during recovery suggest common underlying mechanisms during recovery. Finally, changes in all the above-mentioned parameters of the ribcage muscles could be accurately assessed non-invasively in healthy adults.

## Ethical approval

The study was conducted in accordance with the guidelines of the Declaration of Helsinki, and approved by the ethics committee of the Hospital Universitário Onofre Lopes (HUOL/EBSERH—Brazil) under number 3.084.956.

## Supplementary Information

Supplementary Information 1.

Supplementary Information 2.

Supplementary Information 3.

Supplementary Information 4.

Supplementary Information 5.

Supplementary Information 6.
