# Supplementary material for: Changes in electromyographic activity, mechanical power, and relaxation rates following inspiratory ribcage muscle fatigue
Source: Sci Rep. 2021 Jun 14;11:12475. doi: 10.1038/s41598-021-92060-y (PMC8203654; doi:10.1038/s41598-021-92060-y)
Supplement: Supplementary file 1 — Supplementary Information 1. [file 41598_2021_92060_MOESM1_ESM.docx]

**Changes in electromyographic activity, mechanical power, and relaxation rates following inspiratory ribcage muscle fatigue**

**Scientific Reports**

Antonio Sarmento^1^, Guilherme Fregonezi^1,*^, Maria Lira^1^, Layana Marques^1^, Francesca Pennati^2^, Vanessa Resqueti^1^, Andrea Aliverti^2^

^1^PneumoCardioVascular Laboratory - Hospital Universitário Onofre Lopes, Empresa Brasileira de Serviços Hospitalares (EBSERH) & Laboratório de Inovação Tecnológica em Reabilitação, Departamento de Fisioterapia, Universidade Federal do Rio Grande do Norte, Natal, Brazil.

^2^Dipartimento di Elettronica, Informazione e Bioingegneria, Politecnico di Milano, Milan, Italy.

*Corresponding author: [*fregonezi.guilherme@gmail.com*](mailto:fregonezi.guilherme@gmail.com)

**SUPPLEMENTARY INFORMATION IN DEPTH OF METHODS, STATISTICAL ANALYSIS AND RESULTS**

**STATISTICAL ANALYSIS**

Changes in end-expiratory chest wall volume during recovery were compared with pre-fatigue values using Friedman’s test and two-stage false discovery rate test correction (threshold value of 5%). Regarding %RMS during the fatigue protocol, data from every 50 seconds was averaged (i.e., 0 to 50, 51 to 100, 101 to 150, and 151 to 200 seconds), and ordinary One-way ANOVA or Kruskal–Wallis test was applied to compare %RMS change between muscles. In the event of statistical significance, Bonferroni’s or Dunn’s post hoc test was used, respectively, to identify differences between muscles.

|  | Pre | 1^st^ | 2^nd^ | 3^rd^ | 4^th^ | 5^th^ | 6^th^ | 7^th^ | 8^th^ | 9^th^ | 10^th^ | Mean rec. |
| --- | --- | --- | --- | --- | --- | --- | --- | --- | --- | --- | --- | --- |
| Pressure | 21.37 | 21.00 | 18.29 | 20.93 | 24.50 | 20.84 | 23.34 | 24.20 | 21.90 | 24.39 | 24.32 | 22.37 |
| TPS | 10.22 | 15.83 | 26.51 | 22.34 | 25.37 | 17.97 | 20.99 | 22.84 | 28.14 | 22.80 | 16.90 | 21.96 |
| CT | 15.49 | 22.19 | 23.59 | 13.10 | 16.80 | 14.36 | 15.84 | 13.54 | 13.24 | 16.16 | 9.908 | 15.87 |
| MRDP | 12.74 | 19.34 | 14.84 | 16.41 | 18.31 | 18.30 | 17.21 | 17.08 | 14.73 | 20.12 | 15.13 | 17.14 |
| MRPD/MRR | 22.78 | 21.95 | 29.98 | 26.26 | 26.85 | 27.34 | 28.72 | 20.98 | 21.72 | 21.45 | 24.45 | 24.97 |
| MRR | 22.38 | 24.38 | 27.95 | 24.09 | 20.59 | 22.37 | 22.14 | 25.34 | 22.04 | 20.09 | 20.98 | 22.99 |
| ½RT | 17.16 | 20.73 | 24.55 | 15.46 | 15.26 | 13.33 | 17.68 | 16.08 | 19.25 | 15.90 | 15.99 | 17.42 |
| τ | 24.38 | 28.87 | 27.83 | 23.79 | 35.91 | 22.40 | 22.25 | 25.18 | 26.23 | 26.06 | 31.92 | 27.04 |

**Supplementary information 1. Coefficients of variation of SNIP parameters between subjects.**

Values are presented as percentage. TPS: time to peak shortening; CT: contraction time; MRPD: maximum rate of pressure development; MRPD/Peak: maximum rate of pressure development normalized by peak pressure; MRR: maximum relaxation rate; τ : time constant of pressure decay (tau); ½RT: half-time of the relaxation curve. Last column represents mean of coefficient of variation of SNIP parameters during recovery.

**Supplementary information 2.**

*Calculation of the slopes of the exponential regression lines*

All exponential curves that fitted values of the present study reached a plateau. In this sense, the equation that best describes this behavior during the fatigue protocol (decay) is expressed as follows:

y = (y_0_ - a) e^-k·t^ + a, (E1)

where “*y*” is any value in the y-axis; “*t*” is time in the x-axis; “*y_0_*” is the value in the *y*-axis when *t* equals to “zero”; “*a*” is the value of the y-axis at high *t* values (asymptotic value); and “*k*” is the rate constant value expressed as the inverse value of the x-axis.

To allow comparisons between slopes of linear and exponential regressions, the time derivative of the equation E1 was calculated at the beginning of the fatigue protocol (*t* = 0) as follows:

$\left. \frac{dY}{dt} \right|{}_{t=0}$= -k (y_0_ - a) (E2).

On the other hand, the following exponential growth equation fits the values during the recovery:

y = y_0_ + (a - y_0_) (1 - e^-k·t^) (E3).

The derivative of E3 at time zero equals to:

$\left. \frac{dY}{dt} \right|{}_{t=0}$= k (a - y_0_) (E4).

**Supplementary information 3.**

**
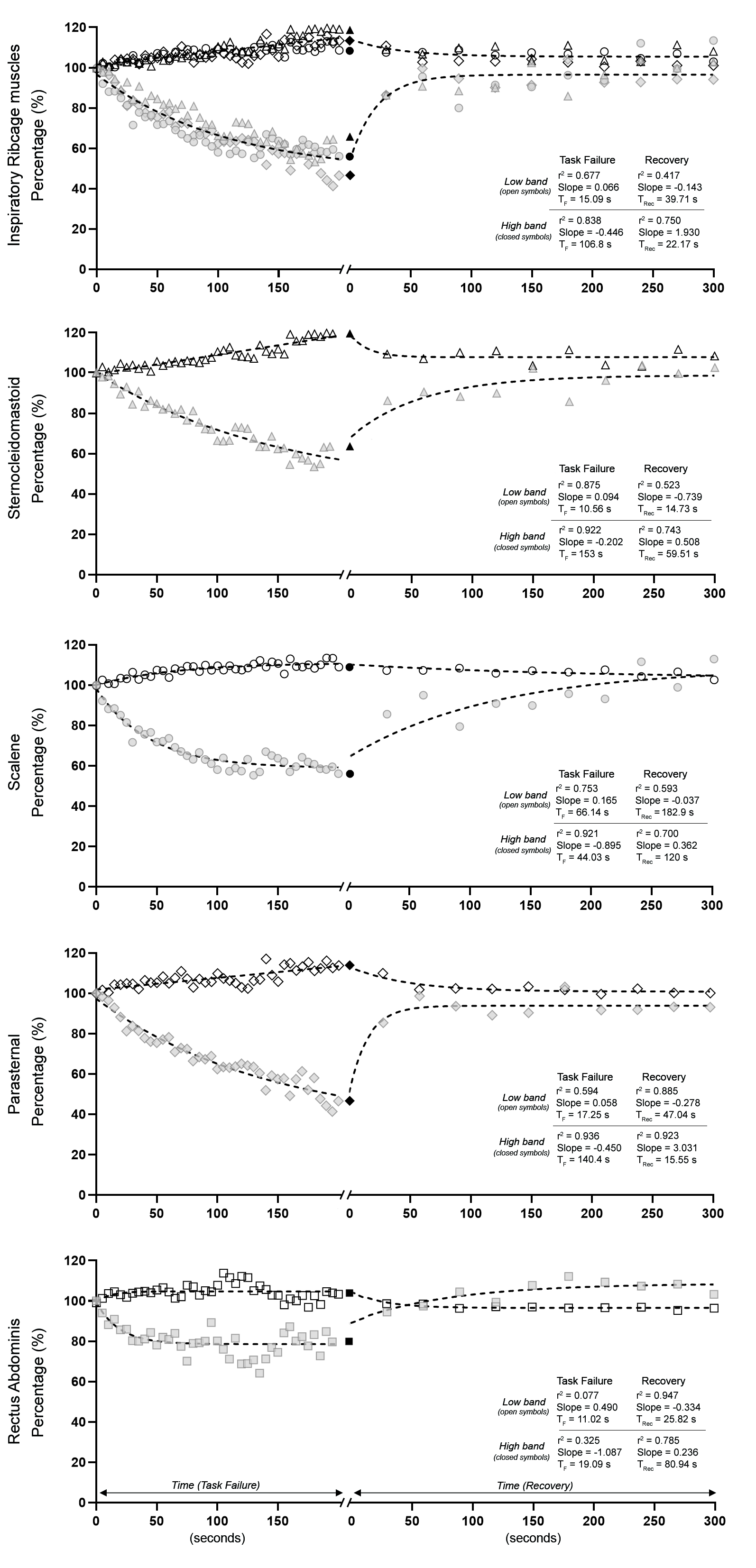
**

**
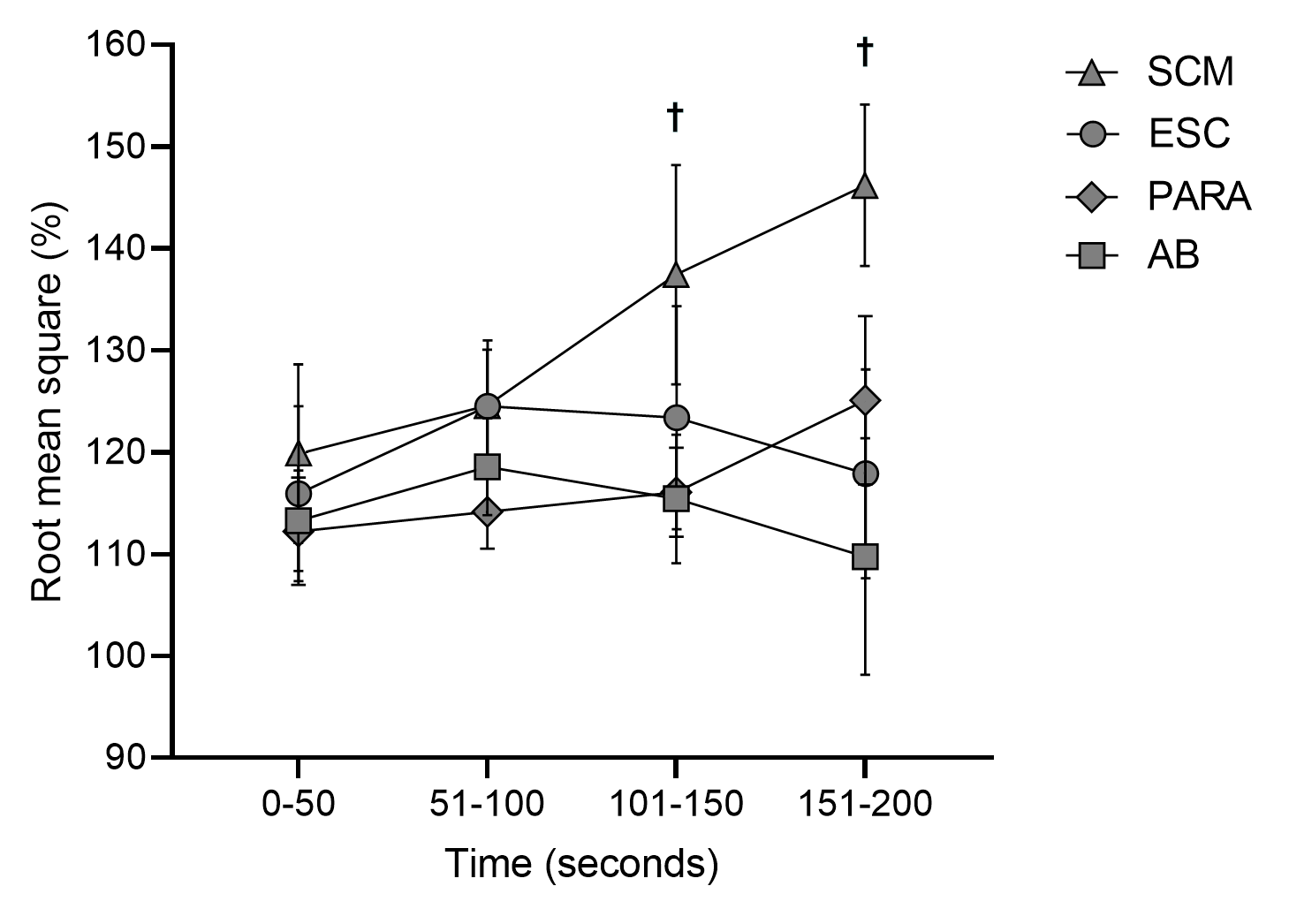
Supplementary information 4.**

**Supplementary information 5. Regression equations that fitted the power spectrum parameters during fatigue and recovery.**

|  |  | **Fatigue protocol** | **Recovery** |
| --- | --- | --- | --- |
| **Median Frequency** | Inspiratory ribcage muscles | *y* = -0.155·*t* + 99.9 | *y* = 60.60 + (104.5-60.6)(1-e^-0.08080·^*^t^*) |
|  | Sternocleidomastoid | *y* = -0.161·*t* + 100.2 | *y* = 60.99 + (102.9-61)(1-e^-0.08879·^*^t^*) |
|  | Scalene | *y* = -0.153·*t* + 99 | *y* = 60.41 + (105.6-60.4)(1-e^-0.1468·^*^t^*) |
|  | Parasternal | *y* = -0.153·*t* + 98.3 | *y* = 60.25 + (105-60.2)(1-e^-0.06462·^*^t^*) |
|  | Rectus abdominis | *y* = (100.9-88.49)e^-0.03884·^*^t^* + 88.49 | *y* = 88.39 + (109.7-88.4)(1-e^-0.03815·^*^t^*) |
| **High/Low ratio** | Inspiratory ribcage muscles | *y* = (95.6-45.34)e^-0.01073·^*^t^* + 45.34 | *y* = 53.3 + (93.95-53.3)(1-e^-0.01966·^*^t^*) |
|  | Sternocleidomastoid | *y* = (99.25-25.26)e^-0.00561·^*^t^* + 25.26 | *y* = 52.38 + (100.5-52.38)(1-e^-0.02278·^*^t^*) |
|  | Scalene | *y* = (98.08-56.68)e^-0.02536·^*^t^* + 56.68 | *y* = 0.138·*t* + 67.22 |
|  | Parasternal | *y* = (91.99-36.86)e^-0.00809·^*^t^* + 36.86 | *y* = 46.62 + (82.44-46.62)(1-e^-0.03149·^*^t^*) |
|  | Rectus abdominis | *y* = (99.09-76.29)e^-0.05595·^*^t^* + 76.29 | *y* = 78.69 + (107.2-78.69)(1-e^-0.01394·^*^t^*) |
| **High band** | Inspiratory ribcage muscles | *y* = (96.59-47.03)e^-0.00935·^*^t^* + 47.03 | *y* = 53.57 + (96.38-53.57)(1-e^-0.0451·^*^t^*) |
|  | Sternocleidomastoid | *y* = (99.92-40.91)e^-0.00653·^*^t^* + 40.91 | *y* = 68.5 + (98.77-68.5)(1-e^-0.0168·^*^t^*) |
|  | Scalene | *y* = (98.29-58.85)e^-0.02271·^*^t^* + 58.85 | *y* = 65.64 + (109.1-65.64)(1-e^-0.00833·^*^t^*) |
|  | Parasternal | *y* = (97.32-34.07)e^-0.00712·^*^t^* + 34.07 | *y* = 47.89 + (95-47.89)(1-e^-0.06432·^*^t^*) |
|  | Rectus abdominis | *y* = (99.35-78.59)e^-0.05328·^*^t^* + 78.59 | *y* = 88.87 + (108-88.87)(1-e^-0.01235·^*^t^*) |
| **Low band** | Inspiratory ribcage muscles | *y* = -0.067·*t* + 101.7 | *y* = (114.5-105.8)e^-0.02519·^*^t^* + 105.8 |
|  | Sternocleidomastoid | *y* = -0.094·*t* + 99.58 | *y* = (119-108.1)e^-0.06787·^*^t^* + 108.1 |
|  | Scalene | *y* = 100.4 + (111.4-100.4)(1-e^-0.01512·^*^t^*) | *y* = (111.3-104.4)e^-0.00546·^*^t^* + 104.4 |
|  | Parasternal | *y* = -0.058·*t* + 102.1 | *y* = (115.5-102.4)e^-0.02126·^*^t^* + 102.4 |
|  | Rectus abdominis | *y* = 100.3 + (105.7-100.3)(1-e^-0.09075·^*^t^*) | *y* = (104.9-96.27)e^-0.03873·^*^t^* + 96.27 |

Linear (*y = a·t + y_0_*) (Equation 1), exponential decay [*y = (y_0_-a) e^-k^*^·^*^t^ + a*] (Equation 2), and exponential growth [*y = y_0_ + (a - y_0_) (1-e^-k^*^·^*^t^)*] (Equation 3) equations fitted the power spectrum parameters in different moments. “*y”* is any power spectrum parameter in the *y*-axis; “*t*” is time in the *x*-axis; “*y_0_*” is the power spectrum parameter when *t* equals to zero; “*a*” is the value of the *y*-axis at high *t* values (asymptotic value); and “*k*” is a constant value expressed in the inverse value of the *x*-axis. For **median frequency**, Equation 1 fitted all values during the fatigue protocol but the RA, while Equation S3 fitted the recovery values. For **high/low ratio** values, Equation 2 fitted the values during the fatigue protocol; while Equation S1 fitted the values of the scalene, and Equation S3 fitted the values of the inspiratory ribcage, sternocleidomastoid, parasternal and rectus abdominis muscles during recovery. For the **high band** of the power spectrum, Equation 2 fitted the values of all muscles during the fatigue protocol, and Equation S3 fitted the recovery values. For the **low band** of the power spectrum, Equation 1 fitted the values of the inspiratory ribcage muscles, sternocleidomastoid, and parasternal muscles, while Equation 3 fitted the values of the scalene and rectus abdominis muscles during the fatigue protocol. Equation 2 fitted all muscles during the recovery moment.

**Supplementary information 6.**

**
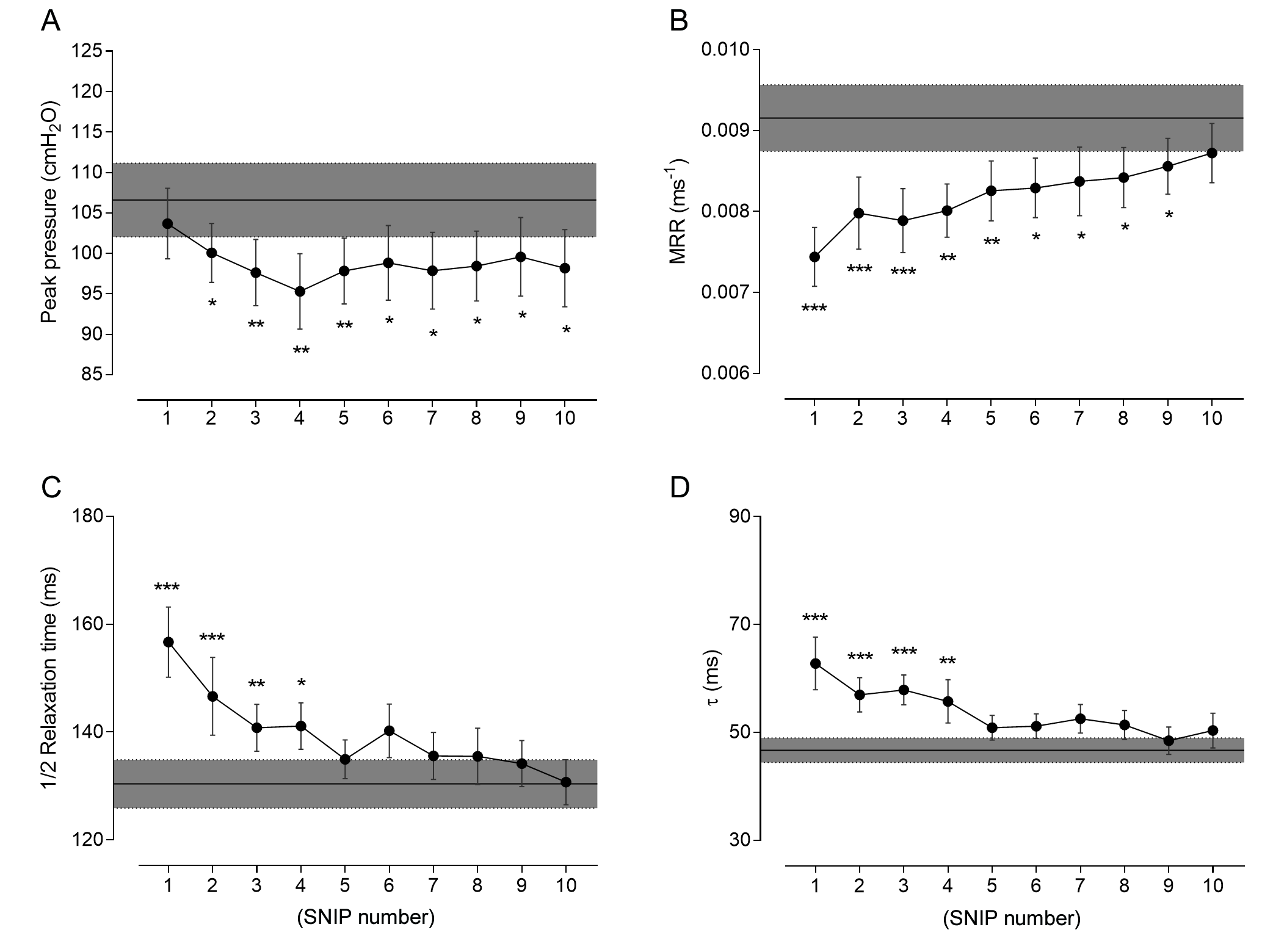
**

**Supplementary information 7.**

**
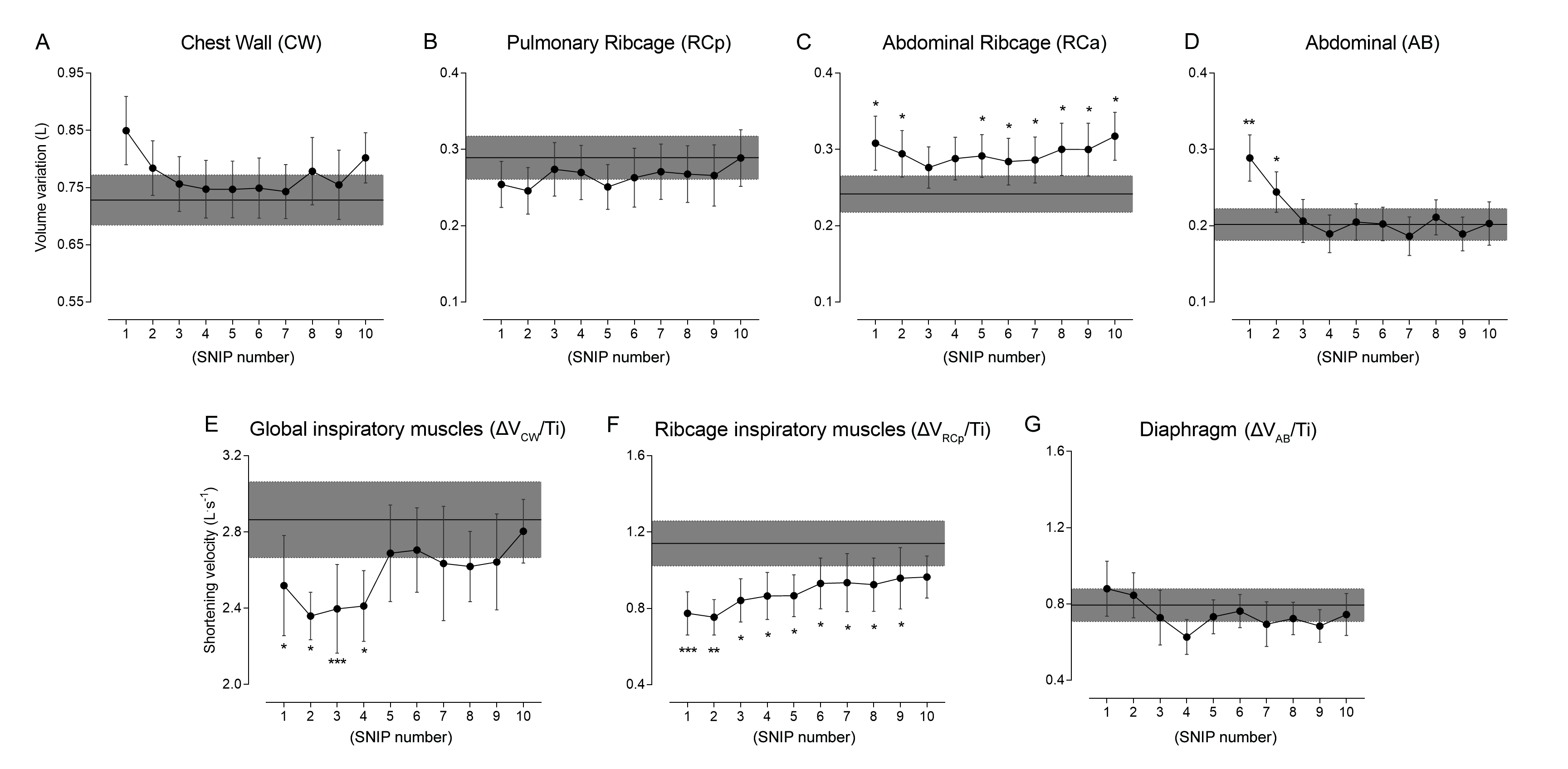
**

**Supplementary information 8.**

**
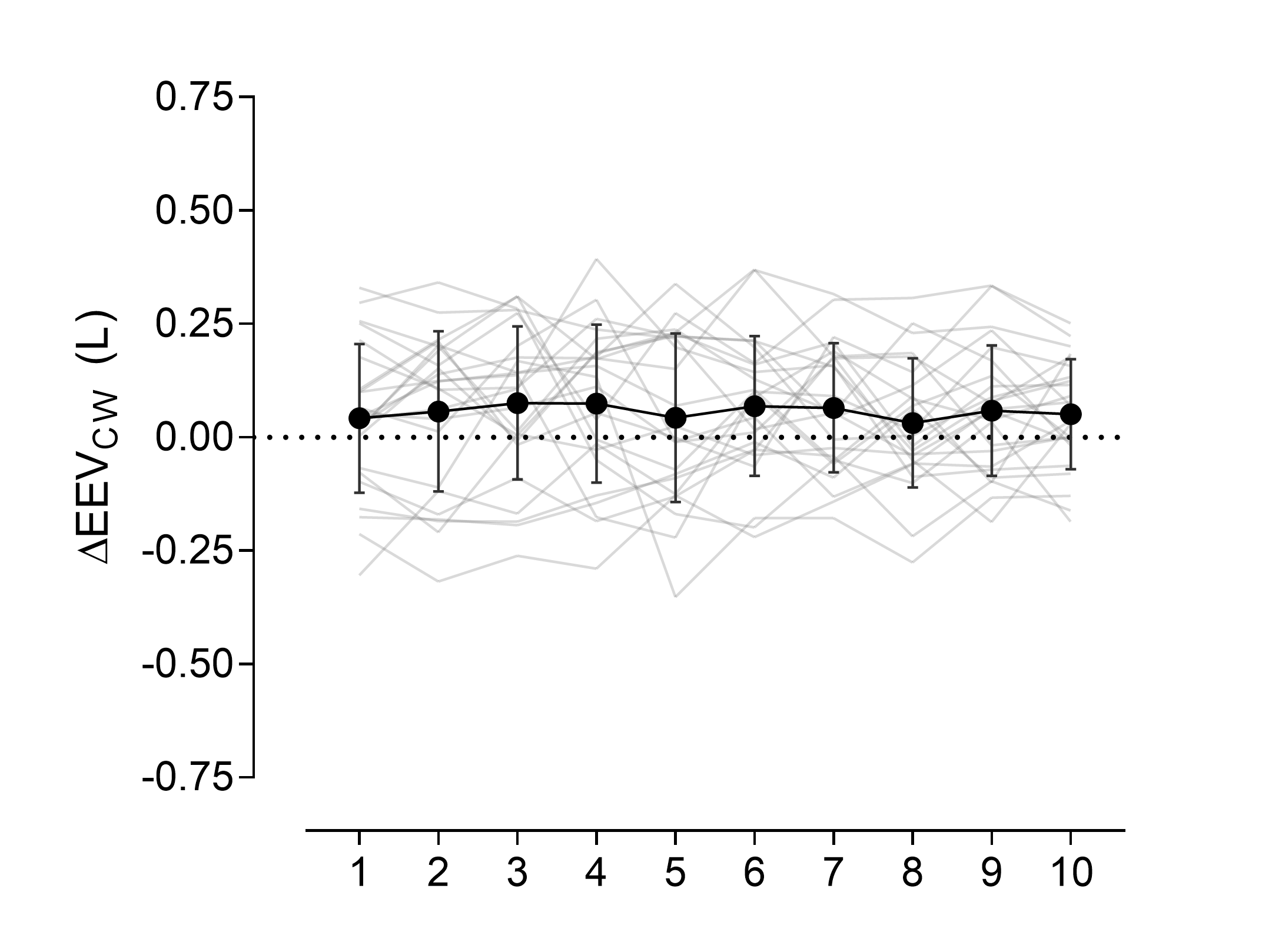
**

**Supplementary information 9.**

**
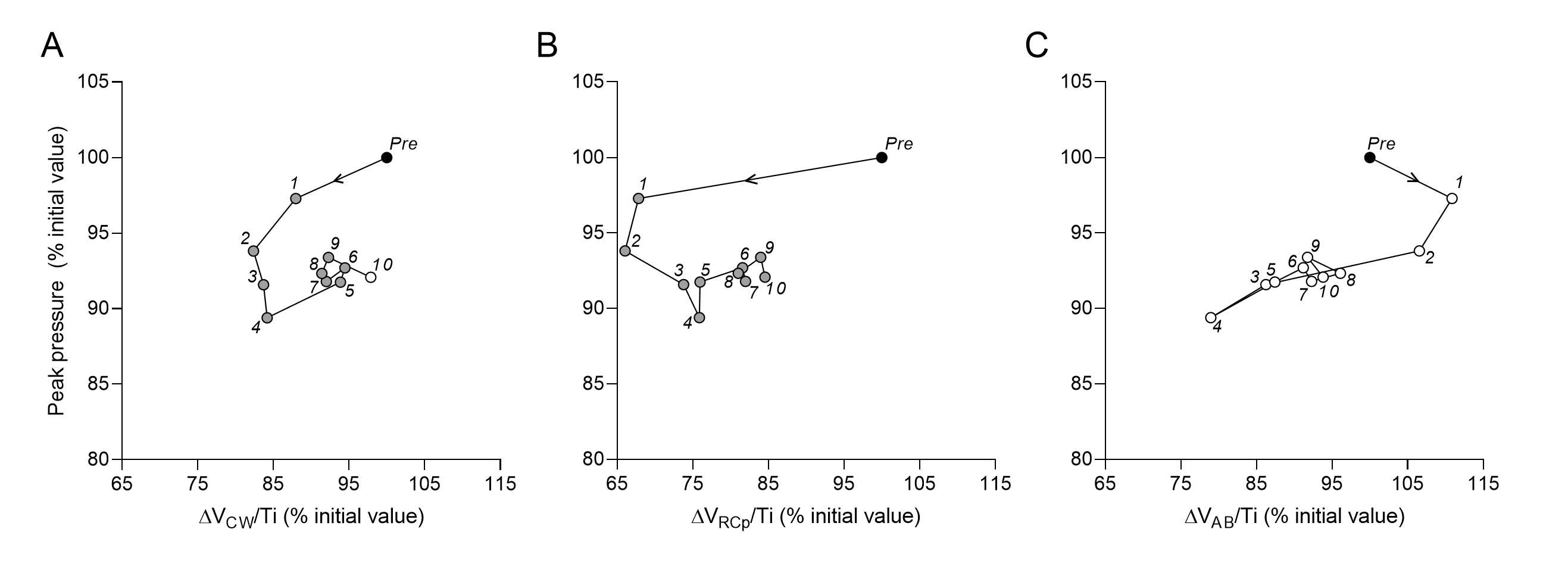
**

**Supplementary information 10.**

**
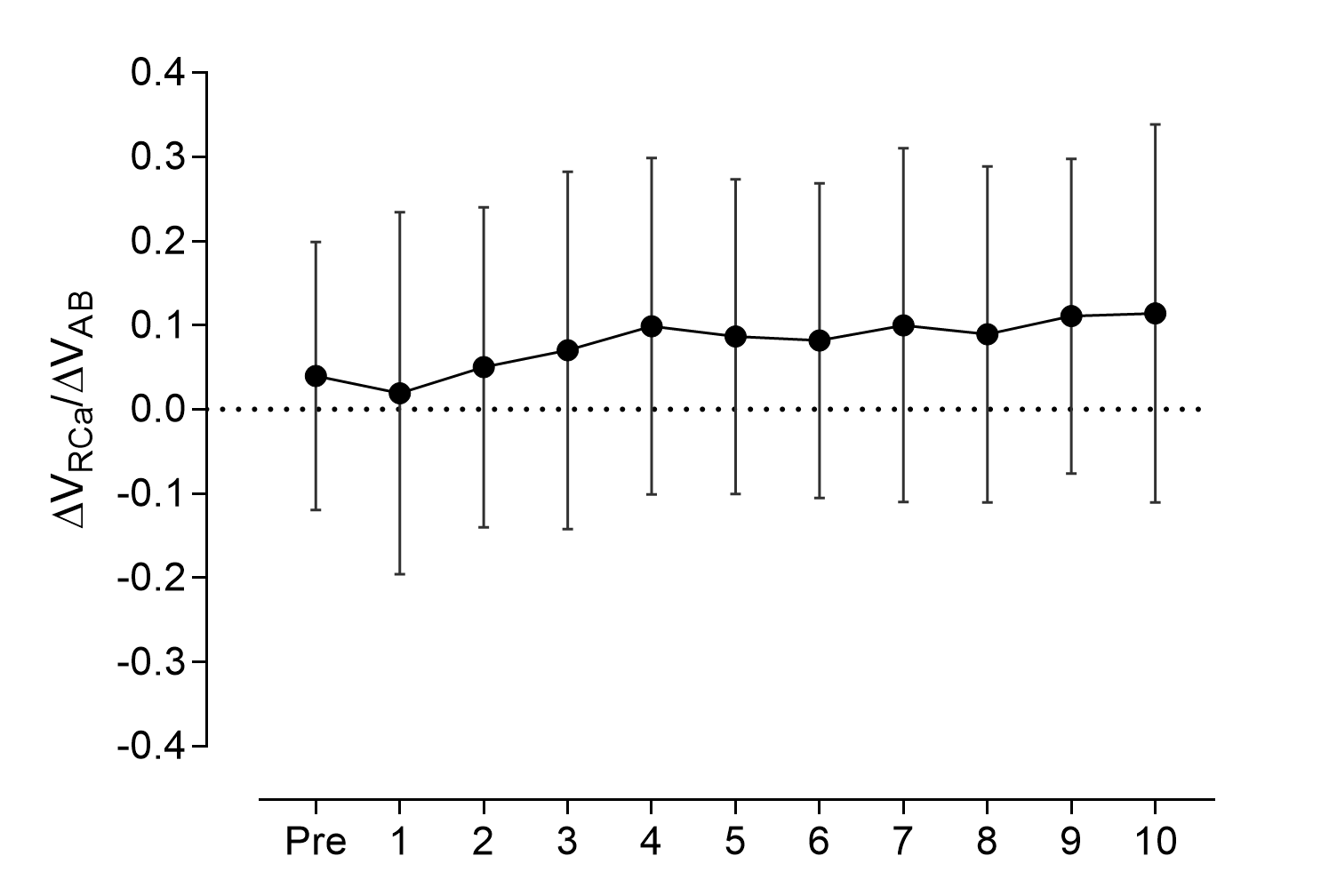
**

**LEGENDS TO SUPPLEMENTARY INFORMATION**

**Supplementary information 1.** Coefficients of variation of SNIP parameters between subjects.

**Supplementary information 2.** Calculation of the slopes of the exponential regression lines.

**Supplementary information 3**. Time courses of the normalized high (grey symbols) and low (white symbols) frequency bands of the power spectrum of inspiratory ribcage muscles (mean values of three muscles studied) during fatigue protocol and recovery moments, as well as each muscle separately. Each point during the fatigue protocol (grey symbols) represent data averaged from 5 seconds, while points during recovery (black symbols) represent data extracted from each SNIP maneuver (i.e., ten SNIP maneuvers with an interval of 30 s in between). All variables are normalized to their initial value during the fatigue protocol. In each muscle, the starting point at time zero (black symbols) corresponds to the last point of the fatigue protocol. Triangles, circles, and diamonds represent the sternocleidomastoid, scalene, and parasternal muscles. Lines represent the regression curves that fitted maximum values in a least-square sense, and equations are shown in Supplementary information 4. T_F_ and T_Rec_ represent time constant during the fatigue protocol and recovery, respectively. %: percentage; s: seconds.

**Supplementary information 4**. Comparison of %RMS changes between sternocleidomastoid (SCM - triangles), scalene (ESC - circles), parasternal (PARA - diamonds), and rectus abdominis (RA - squares) muscles during the fatigue protocol. Data were averaged during every 50 seconds of the fatigue protocol and represented as mean±SD values between 0 and 50 s, 51-100 s, 101-150 s, and 151-200 s. All variables are normalized to their initial value during the fatigue protocol. Cross symbols represent statistically significant differences (p<0.0001) between SCM and other muscles studied. %: percentage.

**Supplementary information 5.** Regression equations that fitted the power spectrum parameters during fatigue and recovery.

**Supplementary information 6**. Data are shown as mean±SE of all 26 subjects included in the study. Peak pressure (panel A), maximum relaxation rate (MRR - panel B), half-time of relaxation curve (½RT - panel C), and time constant of the relaxation curve (τ - panel D) obtained from sniff curves of all subjects during recovery were compared with pre-fatigue values (grey bands). Lines inside grey bands represent mean±SE of pre-fatigue values. cmH2O: centimeters of water; ms: milliseconds. *p<0.05; **p<0.01; and ***p<0.0001.

**Supplementary information 7**. Data are shown as mean±SE of all 26 subjects included in the study. Chest wall (panel A) and compartmental [pulmonary ribcage (panel B), abdominal ribcage (panel C), and abdominal (panel D)] volumes, shortening velocity index of the global inspiratory (ΔVCW/Ti– panel E), ribcage (ΔVRCp/Ti – panel F), and diaphragm (ΔVAB/Ti – panel G) muscles obtained during the SNIP maneuvers during recovery compared with pre-fatigue moment (grey bands). Lines inside grey bands represent mean±SE pre-fatigue values. *p<0.05; **p<0.01; and ***p<0.0001.

**Supplementary information 8**. Mean±SD of changes in end-expiratory chest wall volume (∆EEV_CW_), in liters (L), recorded at initiation of SNIP maneuvers during recovery. No significant differences (p=0.721) were observed between recovery and pre-fatigue (line at zero). Grey lines indicate individual values during recovery.

**Supplementary information 9**. Relative mean changes of peak pressure (y-axis) in relation to mean changes in shortening velocity index (x-axis) of global inspiratory (ΔV_CW_/Ti – panel A), ribcage (ΔV_RCp_/Ti –panel B), and diaphragm (ΔV_AB_/Ti – panel C) muscles. Numbers near dots represent the order of SNIP maneuvers during recovery.

**Supplementary information 10.** Means ± SD of the ratio between abdominal volume (V_AB_) and abdominal rib cage volume (V_RCa_) before (Pre) and after the fatigue protocol. No significant changes were observed, indicating that dV_RCa_/dV_AB_ was constant after the fatigue protocol. This confirms that ΔV_AB_/Ti can also be used as an index of velocity of diaphragmatic shortening in the protocol performed.
