# Supplementary figures and images for: Changes in electromyographic activity, mechanical power, and relaxation rates following inspiratory ribcage muscle fatigue
Source: Sci Rep. 2021 Jun 14;11:12475. doi: 10.1038/s41598-021-92060-y (PMC8203654; doi:10.1038/s41598-021-92060-y)

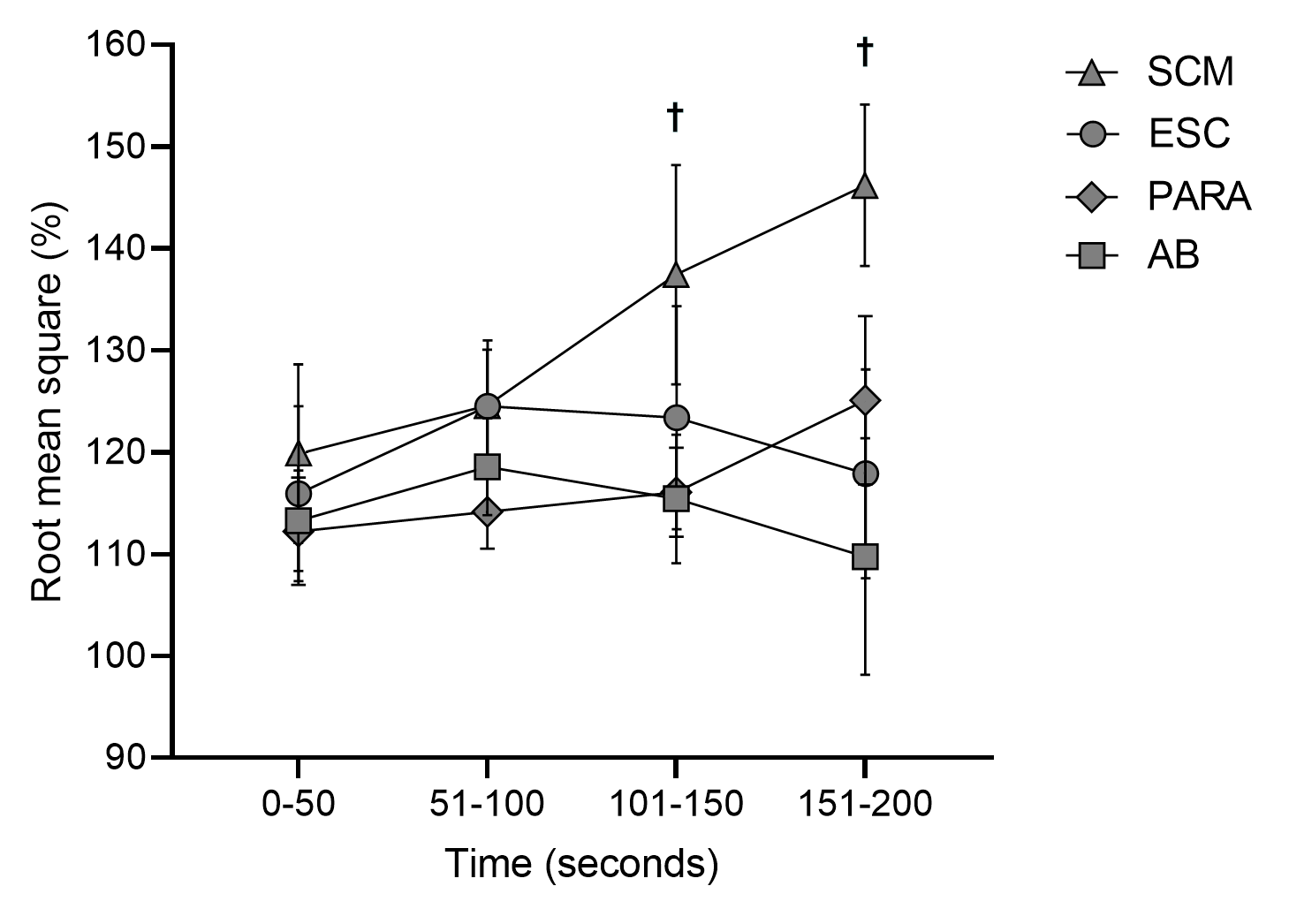

Supplement: Supplementary file 2 — Supplementary Information 2. [file 41598_2021_92060_MOESM2_ESM.tif]

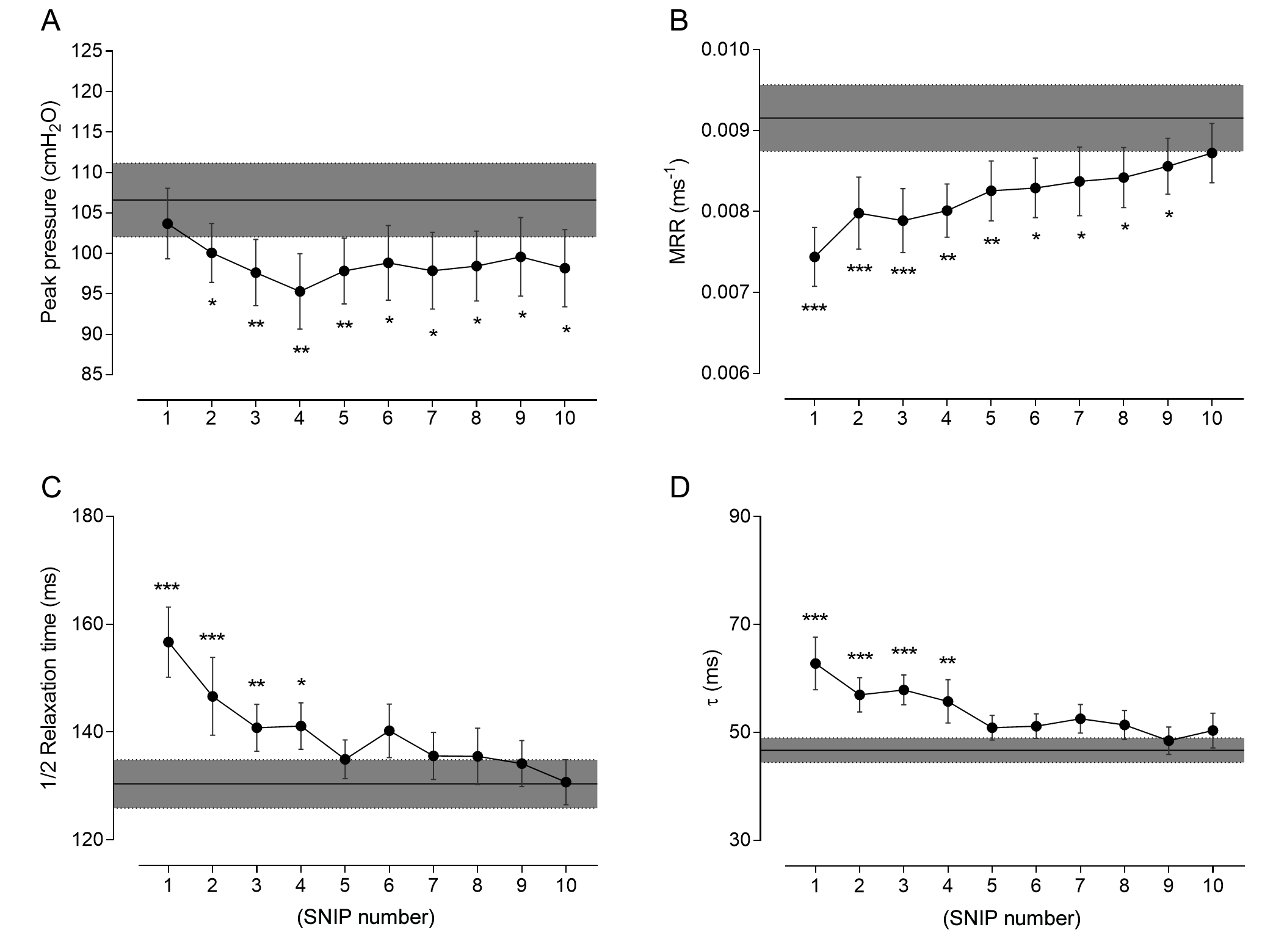

Supplement: Supplementary file 3 — Supplementary Information 3. [file 41598_2021_92060_MOESM3_ESM.tif]

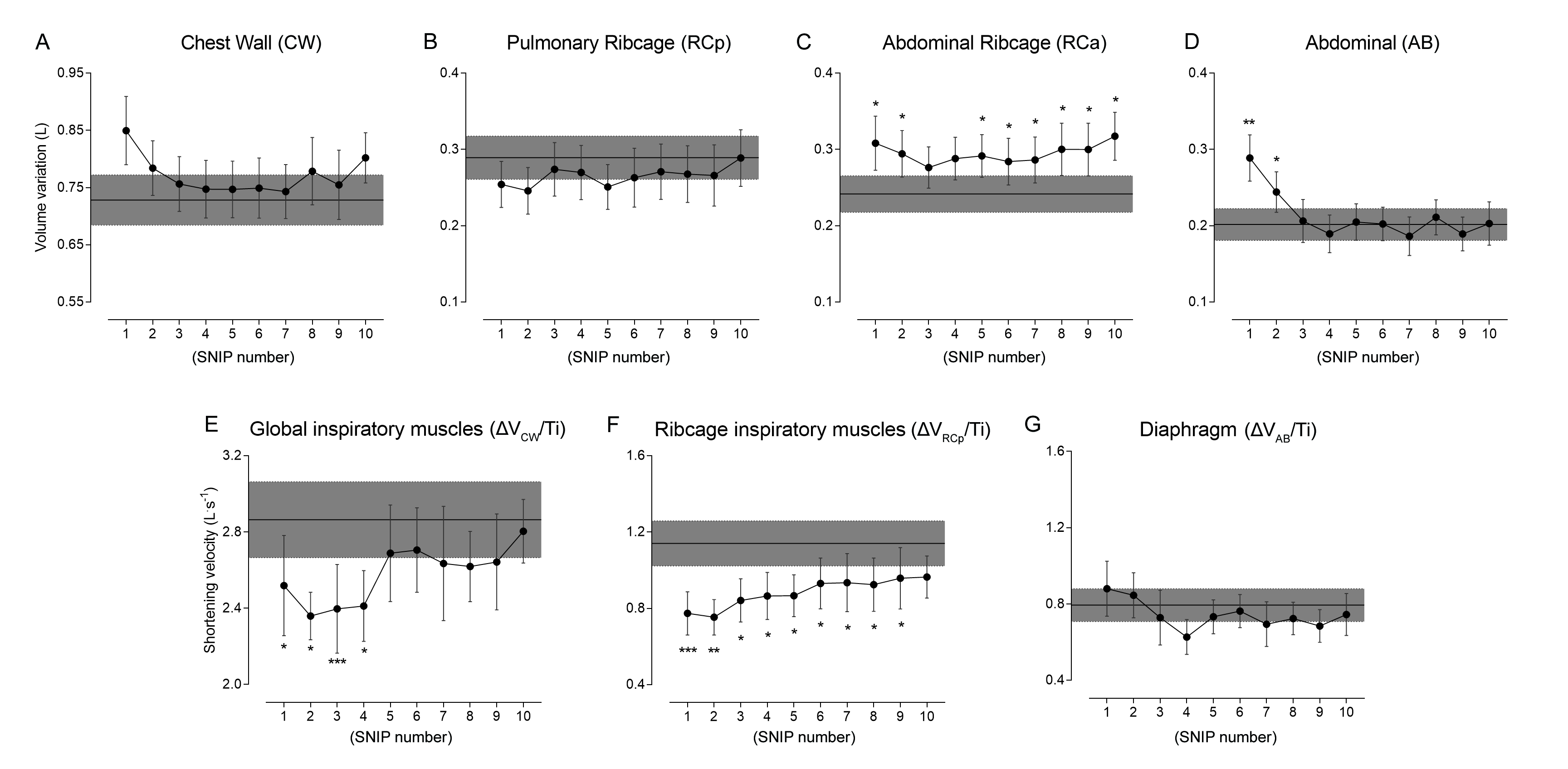

Supplement: Supplementary file 4 — Supplementary Information 4. [file 41598_2021_92060_MOESM4_ESM.tif]

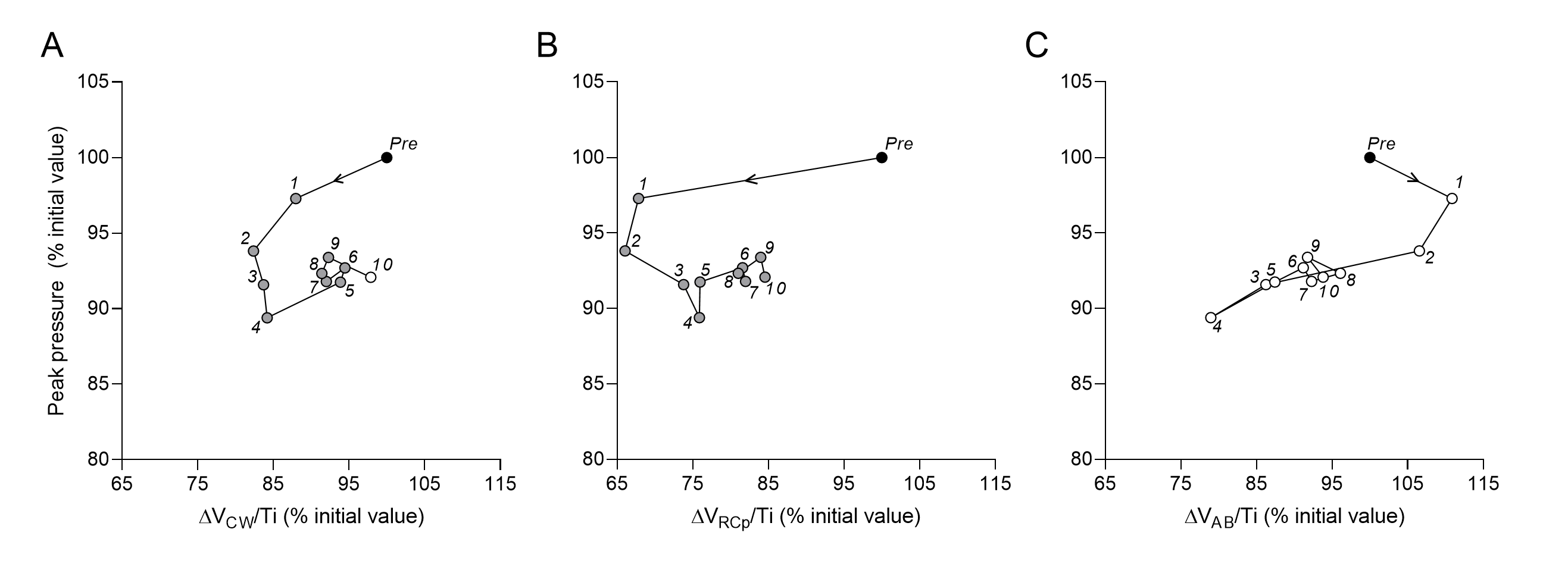

Supplement: Supplementary file 5 — Supplementary Information 5. [file 41598_2021_92060_MOESM5_ESM.tif]

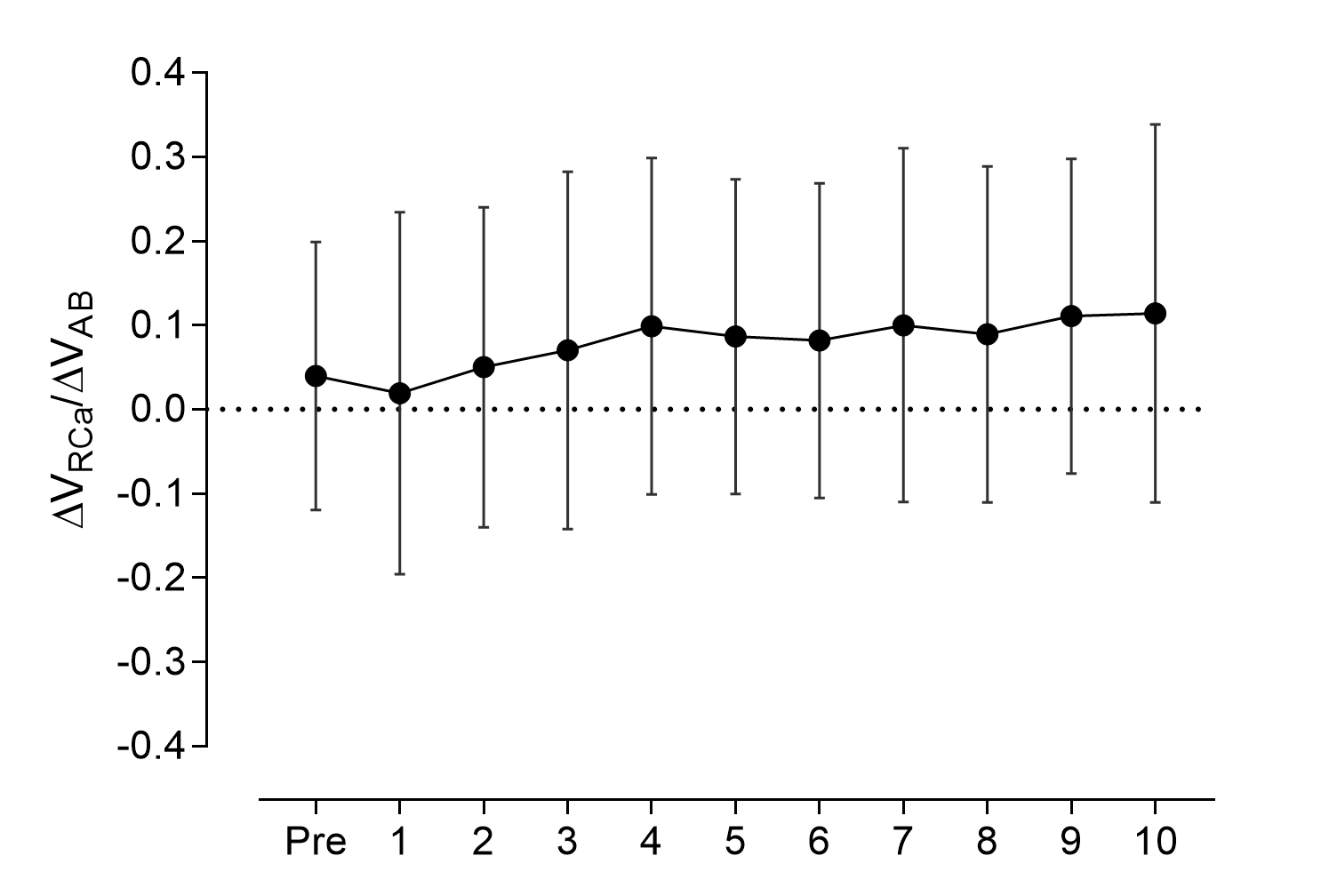

Supplement: Supplementary file 6 — Supplementary Information 6. [file 41598_2021_92060_MOESM6_ESM.tif]
